# Immune Cell Causality and Glucocorticoid Trade-Off in Frozen Shoulder: Integrative MR-RNA-Seq Analysis

**DOI:** 10.3390/biomedicines14081699

**Published:** 2026-07-29

**Authors:** Bing Song, Zhangrong Zhan, Hua Gao, Hui Che, Yuefeng Hao, You Li, Dan Hu

**Affiliations:** 1 Department of Orthopaedics, Suzhou Municipal Hospital, The Affiliated Suzhou Hospital of Nanjing Medical University, Suzhou 215002, China; 2 Gusu School, Nanjing Medical University, Suzhou 215001, China; 3 Department of Orthopaedics, Taizhou People’s Hospital of Nanjing Medical University, Taizhou School of Clinical Medicine, Nanjing Medical University, Taizhou 225300, China

**Keywords:** immune cells, glucocorticoids, frozen shoulder, Mendelian randomization, RNA sequencing

## Abstract

**Objectives:** Frozen shoulder (FS) is characterized by chronic inflammation and fibrosis of the joint capsule, yet the causal roles of immune cell subtypes remain unclear. This study aimed to identify immune cell traits causally associated with FS and evaluate the potential impact of glucocorticoids (GCs) on these immune signatures. **Methods:** A two-sample Mendelian randomization (MR) analysis was performed using genome-wide association study data for 731 immune cell traits and FS. RNA sequencing (RNA-seq) of FS and control capsular tissues was performed to validate immune cell-related signatures and immune infiltration patterns. MR analysis was further used to investigate the relationship between GCs, FS, and immune cell traits. **Results:** MR identified seven immune cell traits as potential risk factors and 17 as potential protective factors in FS. RNA-seq confirmed the up-regulation of five of these traits, which positively correlated with the inflammatory, fibrotic and angiogenic features of FS pathology. Notably, GCs showed no causal association with FS but were negatively associated with a protective immune cell trait. **Conclusions:** Our findings reveal a complex immune cell landscape underlying FS, characterized by both pathogenic and protective immune traits. The integration of MR and RNA-seq analyses highlights potential therapeutic targets and suggests that the immunological consequences of GC therapy warrant further investigation.

## 1. Introduction

Frozen shoulder (FS), also known as adhesive capsulitis (AC), is a common musculoskeletal disorder characterized by progressive shoulder pain and restricted range of motion. It affects approximately 2–5% of the general population, with a higher prevalence in women and a peak incidence between 40 and 60 years of age [1,2]. The term AC emphasizes the underlying pathology of chronic synovial inflammation, capsular fibrosis, and contracture, whereas FS is commonly used as the clinical term despite the two being used interchangeably in the literature. Established risk factors include diabetes mellitus, thyroid dysfunction, metabolic and autoimmune disorders, trauma, and prolonged shoulder immobilization [3].

Clinically, FS is categorized into primary and secondary forms, depending on whether an identifiable precipitating factor is present. The disease typically evolves through overlapping stages, including a painful inflammatory phase, a stiff “frozen” phase with marked motion limitation, and a recovery or “thawing” phase [1]. Early symptoms commonly include insidious onset shoulder pain, nocturnal discomfort, and progressive restriction of both active and passive movement, particularly external rotation [4]. The freezing phase may last from several weeks to several months and is generally considered the period during which anti-inflammatory interventions, including corticosteroid therapy, are most effective.

FS is increasingly recognized as a chronic inflammatory and fibrotic disorder of the joint capsule, characterized by persistent synovial inflammation, immune cell infiltration, fibroblast activation, and extracellular matrix (ECM) remodeling [5]. Pro-inflammatory cytokines and profibrotic mediators drive fibroblast activation, excessive ECM deposition, and capsular contracture, while pathological angiogenesis sustains immune cell recruitment and chronic inflammation [6,7,8,9]. These interconnected processes establish a self-perpetuating inflammatory–fibrotic cycle that ultimately leads to progressive joint stiffness.

Although multiple inflammatory and matrix-associated molecules (including IL (Interleukin)-6, TNF (Tumor Necrosis Factor)-α, TGF (Transforming Growth Factor)-β1, MMPs (Matrix Metalloproteinases), and collagen derivatives) have been proposed as candidate biomarkers, no validated biomarker has yet entered routine clinical practice. The absence of reliable diagnostic or prognostic biomarkers underscores a critical unmet clinical need in FS.

FS is characterized by a heterogeneous immune microenvironment involving macrophages, T and B lymphocytes, dendritic cells, and mast cells [5]. These immune cell populations play distinct roles in inflammation, fibrosis, angiogenesis, and immune regulation, suggesting that individual immune subsets may exert divergent effects on disease progression [10,11,12]. However, the causal contribution of specific immune cell traits to FS remains unclear.

Mendelian randomization (MR) enables causal inference by using genetic variants as instrumental variables while reducing confounding and reverse causation [13]. Although previous MR studies have identified immune cell traits associated with FS susceptibility, the lack of tissue-level validation limits their biological interpretation [14,15]. Integrating MR with transcriptomic profiling provides an opportunity to link genetic evidence with local immune alterations in FS.

Transcriptomic profiling provides complementary insights by capturing gene expression changes within diseased tissues [16,17]. RNA-sequencing (RNA-seq) of FS capsule samples allows for the identification of immune-related signatures and dysregulated pathways associated with disease progression [18]. However, transcriptomic data alone cannot establish causality. Therefore, integrating MR-based causal inference with tissue-level transcriptomic validation offers a complementary strategy to link genetic predisposition with local immune activity in FS.

Current management of FS includes physiotherapy, home-based stretching exercises, oral non-steroidal anti-inflammatory drugs (NSAIDs), intra-articular corticosteroid injections, hydrodilatation, manipulation under anesthesia, and arthroscopic capsular release in refractory cases [19]. Among these, corticosteroid injections are widely recommended for short-term pain relief and functional improvement [20].

Glucocorticoids (GCs) remain a cornerstone of pharmacological management due to their potent anti-inflammatory and immunomodulatory effects. They act primarily through inhibition of NF-κB and AP-1 signaling pathways, thereby suppressing cytokine production and synovial inflammation [21,22]. However, their long-term disease-modifying effects remain controversial, and concerns persist regarding systemic adverse effects such as metabolic dysregulation and osteoporosis [23]. Importantly, whether immune suppression induced by glucocorticoids modifies the underlying fibroinflammatory process in FS remains insufficiently understood.

In this study, we integrate large-scale Mendelian randomization with transcriptomic profiling of surgical FS capsule biopsies to (i) identify immune cell traits causally associated with FS, (ii) define their local tissue enrichment and pathogenic pathways, and (iii) evaluate the potential impact of glucocorticoid exposure on immune-related traits. By integrating genetic causal inference with tissue-level validation, this study provides a multi-layered framework to elucidate immune cell-specific mechanisms in FS and their therapeutic modulation. Collectively, our findings offer new insight into the immunobiological architecture of FS and support a precision medicine-oriented approach for future therapeutic development.

## 2. Methods

**Study design and GWAS data sources**

Genome-wide association studies (GWAS) provide genetic variants associated with complex traits that can serve as instrumental variables for Mendelian randomization (MR), enabling causal inference with reduced susceptibility to confounding and reverse causation compared with conventional observational studies [24].

The validity of MR analysis relies on three core assumptions: (1) relevance, whereby the selected genetic variants are strongly associated with the exposure trait; (2) independence, whereby the instrumental variables are not associated with potential confounding factors; and (3) exclusion restriction, whereby the genetic variants influence the outcome only through the exposure and not through alternative biological pathways.

**Figure 1:**
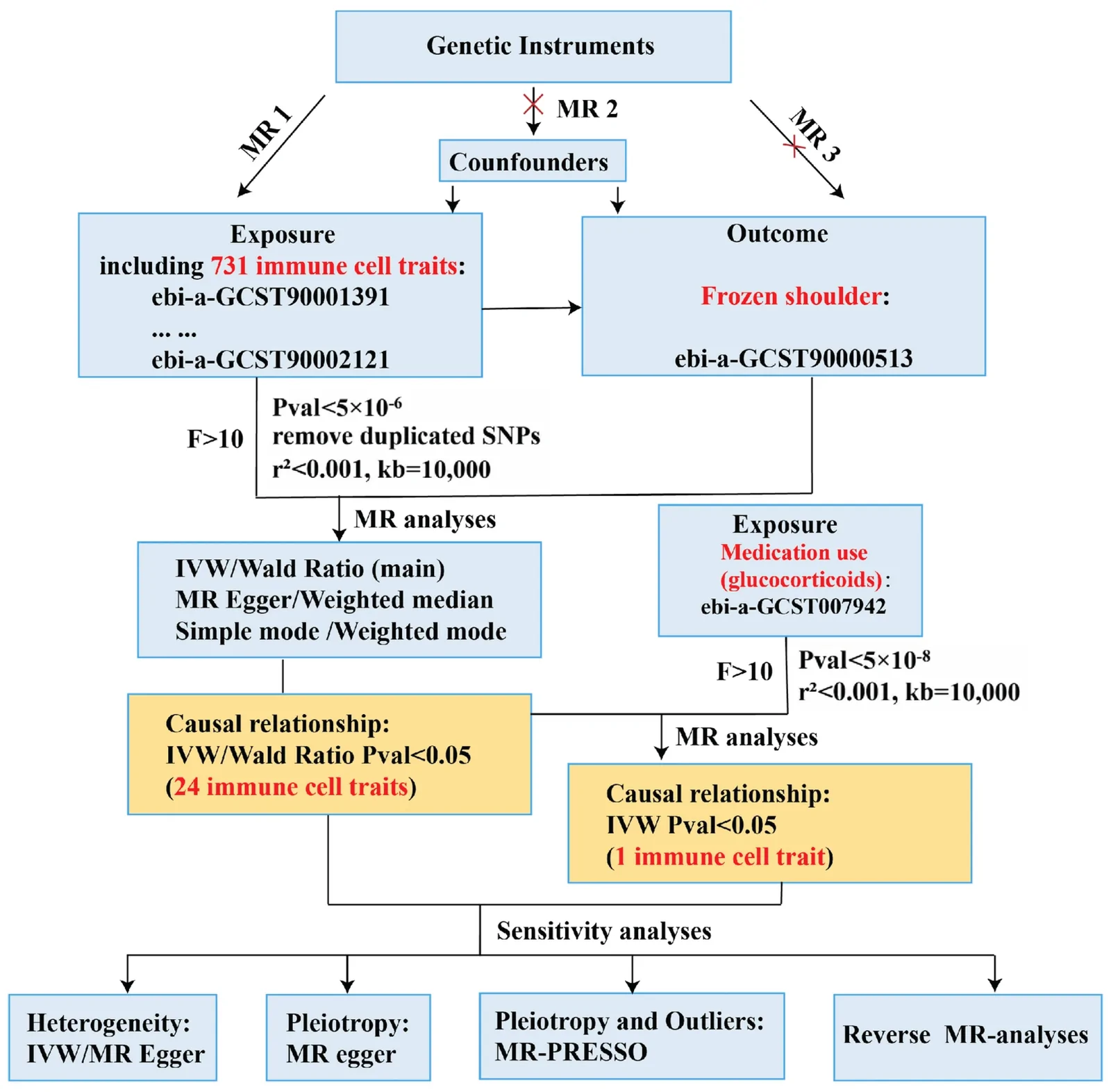
The flowchart of MR study design and analysis methods.

To investigate the causal relationship between 731 immune cell traits and FS, a two-sample MR approach was utilized. Additionally, this MR analysis method was employed to assess the causal relationship between GC and FS as well as the 24 positive immune cell traits identified. To establish credible causality, instrumental variables (IVs) need to strictly adhere to the three main assumptions of MR (Figure 1) [25].

We used GWAS summary statistics data from the GWAS Catalog (GCST90001391–GCST90002121), encompassing 731 immune cell traits (https://www.ebi.ac.uk/gwas/). This dataset comprises 389 surface antigen-related median fluorescence intensities (MFIs), 192 relative counts (ratio between various cells), 32 cell morphological parameters (MP), and a total of 118 cell counts. These data came from a group of 3757 Sardinians [14]. The Sardinian immune cell GWAS was selected as the exposure dataset because it is the largest and most widely used GWAS resource for immune cell traits. Its well-characterized founder population provides a reliable genetic resource for MR analyses [26]. The study on FS, identified by the accession number: GCST90000513, analyzed 8,423,455 SNPs across a cohort of 627,998 individuals of European ancestry (https://www.ebi.ac.uk/gwas/) (Supplementary File S1) [27]. In this study, the exposure comprised genetically predicted immune cell traits derived from the immune cell GWAS, including cell abundance, relative cell proportion, morphology, and cell-surface marker expression.

GC measurement GWAS statistics data were obtained from the GWAS Catalog (GCST007942), including 17,352 European ancestry cases and 188,348 European ancestry controls (https://www.ebi.ac.uk/gwas/) [28]. This study entailed a re-analysis of previously collected and published data, thereby obviating the necessity for additional ethical approval.

**Genetic instrument selection**

To fulfill the first MR assumption, which requires that SNPs demonstrate robust associations with exposure traits, only SNPs showing significantly strong correlations with exposure traits were selected (For immune cells as exposure: *p*val < 5 × 10^−6^, and for GC measurement as exposure and reverse MR analysis exposure: *p*val < 5 × 10^−8^). SNPs associated with immune cell traits were selected as instrumental variables when they reached genome-wide significance (*p* < 5 × 10^−8^). To ensure adequate instrument strength and minimize weak-instrument bias, the F statistic was calculated for each SNP, and variants with F > 10 were retained for subsequent analyses.

Concurrently, duplicate SNPs associated with multiple immune cells were excluded to mitigate pleiotropic bias [29,30]. We conducted clumping with a threshold of r^2^ < 0.001 within a 10,000 kb distance, utilizing the linkage disequilibrium (LD) reference panel from the 1000 Genomes Project (EUR) [31]. LD refers to the non-random co-inheritance of nearby genetic variants. The LD reference panel, derived from the European samples of the 1000 Genomes Project, was used to estimate pairwise correlations between SNPs and identify independent variants. This procedure minimizes redundancy among instruments and reduces bias arising from correlated genetic variants. The selected SNPs from the GWAS of outcome traits were then subjected to the extraction of relevant values. Correction or exclusion was applied as needed to palindromic SNPs with uncertain strand orientation and ambiguous SNPs with inconsistent alleles. SNPs demonstrating significant correlations with the outcome, adhering to a *p*val < 5 × 10^−8^, were excluded to meet the third MR assumption (Figure 1). To mitigate the risk of weak instrumental variable bias, defined by an F-statistic < 10, we applied the F-statistic formula (F = β2/se2) to assess the statistical efficacy of SNPs and excluded those demonstrating poor statistical efficacy [32].

**Joint capsule sample collection**

This study adhered to the approval of the Ethics Committee of the Affiliated Suzhou Hospital of Nanjing Medical University (registered number K-2024-058) and the Declaration of Helsinki. Informed consent was obtained from all participants.

Joint capsule samples were collected from five patients with frozen shoulder (FS) and five control subjects undergoing shoulder surgery at the Affiliated Suzhou Hospital of Nanjing Medical University. FS was diagnosed according to established clinical criteria. Patients with other shoulder disorders or systemic inflammatory diseases were excluded, and control subjects had no evidence of FS [4]. Samples were collected during arthroscopic surgery in a sterile environment and promptly preserved in formalin for blocking or RNAlater^®^ (Sigma-Aldrich, St. Louis, MO, USA) to prevent RNA degradation at −80 °C until further processing.

Owing to the limited availability of surgically obtained capsular tissues, the RNA-seq cohort was used as an independent validation dataset rather than a discovery cohort.

**Masson staining**

Masson staining was performed to assess collagen deposition and fibrosis in joint capsule tissue. Representative images were acquired using a light microscope [33]. The joint capsule samples were fixed in 4% paraformaldehyde (PFA) solution and processed for paraffin embedding and sectioning into 5 mm-thick slices. The paraffinized slices underwent deparaffinization, hydration, and hematoxylin and Masson staining (Solarbio, Beijing, China), according to the product manual.

**RNA sequencing and BioSample data**

The control and FS joint capsule samples obtained from patients underwent total RNA extraction. RNA concentration and purity were assessed using a NanoDrop spectrophotometer (Thermo Fisher Scientific, Waltham, MA, USA), while RNA integrity was evaluated using an Agilent 2100 Bioanalyzer (Agilent Technologies, Santa Clara, CA, USA). Samples with satisfactory RNA quality were used for library construction. mRNA was then enriched using Oligo (dT) magnetic beads and fragmented with Fragmentation Buffer. This fragmented mRNA was used to synthesize both single-stranded and double-stranded cDNA, which was purified thereafter. Following this, PCR enrichment was performed to obtain the cDNA library. Library quality was assessed before sequencing. Paired-end sequencing (PE150) was performed on the Illumina NovaSeq platform (Illumina, Inc., San Diego, CA, USA) by GENOME Biotechnology Co., Ltd., Hangzhou, China. After qualifying the constructed library using Qubit 3.0 and Agilent 2100, it was sequenced on a high-throughput platform. Differential expression and immune cell infiltration analyses were subsequently performed using standard bioinformatic pipelines. We have deposited the Raw BioSample data for RNA-seq in the NCBI database, linked to BioProject accession number PRJNA1098710 (https://www.ncbi.nlm.nih.gov/bioproject/, accessed on 20 July 2026), with all 10 samples available for download.

For RNA-seq analyses, differential gene expression was assessed using log_2_ fold change and adjusted *p*-values. Quantitative real-time PCR was not performed, as transcriptomic validation was based directly on RNA sequencing of clinical capsular tissues. Because MR-derived immune cell traits, xCell-inferred immune cell populations, and RNA-seq-derived marker genes reflect different levels of biological inference, they were integrated as complementary approaches rather than direct one-to-one comparisons to identify convergent immune-related signals in FS.

**Integration strategy of MR, RNA-seq, and xCell analyses**

To explore immune mechanisms in FS, we integrated MR-RNA-seq analysis. First, MR analysis was used to identify immune cell traits with potential causal effects on FS using genome-wide association study (GWAS) data. Second, RNA-seq of FS capsular tissues was performed to characterize differential gene expression and immune-related molecular signatures. xCell deconvolution analysis was applied to bulk RNA-seq data to estimate relative immune cell infiltration patterns within FS tissues.

Importantly, these approaches operate at different biological levels. MR-derived immune cell traits represent genetically predicted immune phenotypes, whereas xCell estimates transcriptome-derived immune cell enrichment scores, and RNA-seq identifies gene-level transcriptional changes. Therefore, no direct one-to-one mapping between MR immune cell traits and xCell-inferred cell types was assumed. Instead, we evaluated whether these independent datasets converged on shared biological processes, including inflammation, fibrosis, and angiogenesis in FS.

**Data analyses**

For MR analyses, the Wald ratio estimator was applied when only one instrumental variable (IV) was available, whereas the random-effects inverse variance weighted (IVW) method was used as the primary approach for multiple IVs [34,35]. Weighted Median, Simple Mode, and Weighted Mode methods were performed as complementary analyses, and consistent effect directions across these approaches were considered supportive of causal associations [36].

Sensitivity analyses included Cochran’s Q test for heterogeneity, MR-Egger intercept and MR-PRESSO tests for horizontal pleiotropy, leave-one-out analysis to evaluate the influence of individual SNPs, and reverse MR analysis to assess potential reverse causality [37,38]. Random-effects IVW models were applied when heterogeneity was detected.

For transcriptomic validation, xCell analysis was performed to estimate the relative abundance of 64 immune and stromal cell types based on gene-expression signatures from bulk RNA-seq data. These inferred immune cell profiles were integrated with MR findings to provide tissue-level validation.

RNA-seq data were processed and statistical plots were generated using GraphPad Prism (version 10; GraphPad Software, San Diego, CA, USA). T-tests were employed for comparing two groups of data, with *p* < 0.05 considered statistically significant. R packages circlize (version: 0.4.12) and corrplot (Version: 0.84) were utilized to plot gene-to-gene correlations. For the MR analysis, we applied the “TwoSampleMR” (version 0.5.7) package, the “MRPRESSO” package, and the “plinkbinr” package in RStudio (version 4.3.1). The statistical significance level that was selected was *p* < 0.05. Multiple-testing correction was performed separately within each prespecified family of hypotheses. Associations with a BH-adjusted *p*-value < 0.05 were considered statistically significant after FDR correction. Associations with a raw *p*-value < 0.05 but a BH-adjusted *p*-value ≥ 0.05 were considered suggestive associations. Associations with a raw *p*-value ≥ 0.05 were considered to show no nominal statistical evidence.

## 3. Results

**Exploring the genetically predicted causal effect of immune cell traits on FS**

**Figure 2:**
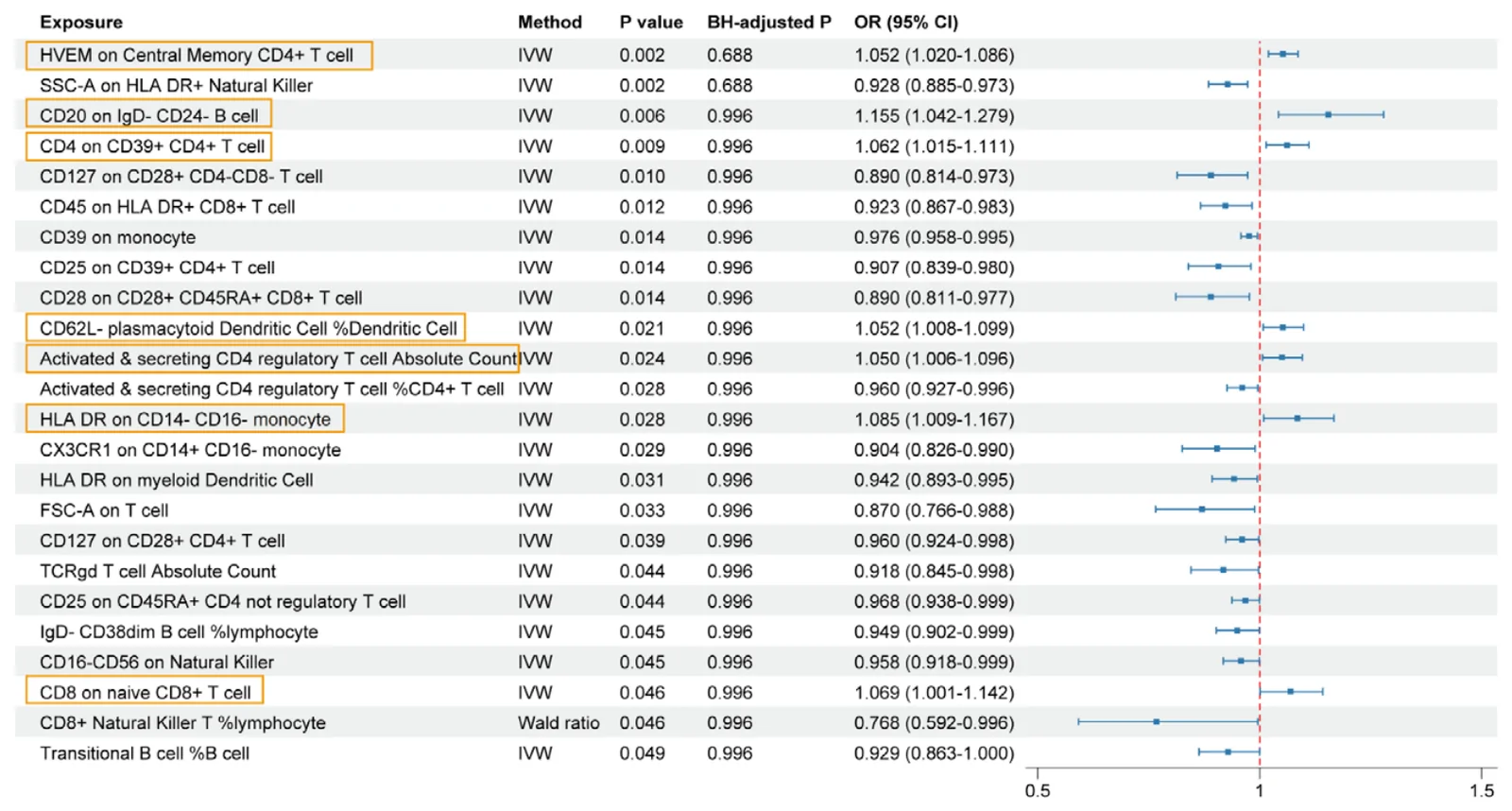
Causal effects of immune cell traits on frozen shoulder identified by Mendelian randomization. Forest plots present odds ratios (ORs) and 95% confidence intervals (CIs) derived from the inverse variance weighting (IVW) or Wald ratio. Orange symbols indicate risk-associated immune cell traits.

We conducted MR analysis to examine the causal relationship between a total of 731 immune cell traits and FS. According to the results of IVW or Wald ratio analysis, we identified 24 immune cell traits that were potentially causally related to FS (Figure 2, Supplementary File S3). Among them, seven immune cell traits showed potential positive associations with an increased risk of FS, including HVEM (Herpesvirus Entry Mediator) on Central Memory CD4+ T cell, CD20 on lgD− CD24− B cell, CD4 on CD39+ CD4+ T cell, CD62L-plasmacytoid Dendritic Cell, Activated and secreting CD4 regulatory T cell Absolute Count, HLA DR on CD14−CD16− monocyte and CD8 on naive CD8+ T cell (highlighted by the box in Figure 2). The remaining 17 immune cell traits showed a negative correlation with the risk of FS.

The OR and 95% CI of their IVW/Wald ratio analysis results are shown in Figure 2. As only one instrumental SNP was available for CD8+ Natural Killer T %lymphocyte, the causal estimate for this trait was calculated using the Wald ratio method, and a conventional MR scatter plot was not applicable. Therefore, scatter plots were generated for the remaining 23 immune cell traits with multiple instrumental SNPs and are provided in Supplementary File S3. The Wald ratio result for CD8+ Natural Killer T %lymphocyte is presented in Supplementary File S2. Each point in the figure represents an SNP. The horizontal axis represents the estimated effect of the SNP on the exposure factor (immune cell traits), and the vertical axis represents the estimated effect of the SNP on the outcome (FS). If the overall slope of these points is positive, it suggests that an increase in exposure will lead to an increase in the risk of the outcome; if the slope is negative, it indicates a protective causal effect. The sensitivity analysis results are presented in Table 1. Heterogeneity tests were performed for immune cell traits with sufficient instrumental SNPs. The Cochrane’s Q test and Rücker’s Q test results for the 20 eligible immune cell traits were all greater than 0.05, indicating no evidence of heterogeneity. For the remaining traits with insufficient instrumental SNPs, heterogeneity tests were not applicable. The results of the MR Egger intercept test and MR-PRESSO test also indicated that there were no pleiotropic effects and outliers. Leave-one-out analysis was used to identify SNPs that might bias causal assessment (Supplementary File S4), demonstrating the robustness of the causal associations between immune cell traits and FS.

To further investigate whether FS leads to dysregulation in the 731 immune cell traits, we conducted reverse MR analyses with FS as exposure and 24 cell traits as outcomes, but the results did not indicate any causal effect (Supplementary Files S5 and S6). Thus, having FS does not necessarily mean that the immune cell traits are abnormal, and there is no reverse causality between them.

**Infiltrated immune cell subtypes based on RNA-seq in FS**

**Figure 3:**
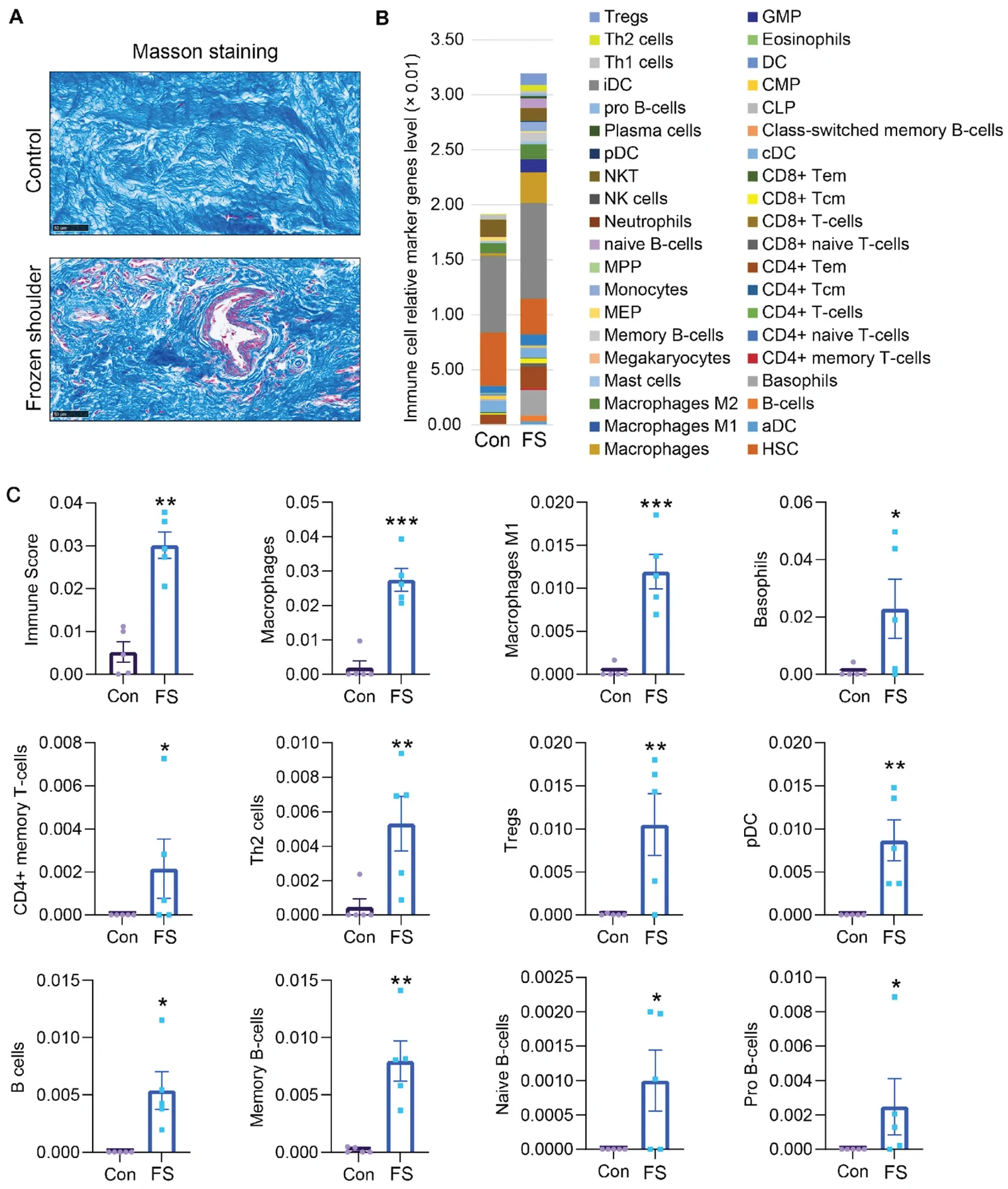
Immune cell relative marker gene expression analysis by RNA-seq. (**A**) Masson staining of normal (control) or frozen shoulder (FS) capsular tissues. Blue represents collagen fibers, while red represents muscle fibers/cytoplasm (red blood cells). (**B**) Comparison of the expression differences of 40 immune cell markers between samples of control and FS groups from RNA-seq. Total expression differences are also depicted in the graph; (**C**) Statistically significant differences (*p* < 0.05) in immune scores and immune cell markers between control and FS groups are presented separately. (*: *p* < 0.05; **: *p* < 0.01; ***: *p* < 0.001).

We collected five normal capsular tissues (control group) from patients with a rotator cuff tear and five adherent tissues from FS patients. Masson staining showed that the collagen arrangement in the control group was relatively orderly and the degree of fibrosis was low. However, the collagen arrangement was disordered and the fibrosis and neovascularization were severe in the FS group (Figure 3A). For these two groups of capsular tissues, we also conducted RNA-seq and used Xcell [18] to finely resolve the heterogeneity of the bulk RNA-seq data, facilitating precise identification and quantitative enrichment of 40 immune cell types. Additionally, we observed a visibly higher relative level of immune cell marker genes in the FS group compared to the control (Figure 3B). The composition of cell subtypes for each sample set is illustrated in Supplementary File S7. Moreover, the immune score calculated from the Xcell indicated higher immune cell infiltration in the FS group than in the control (the first histogram in Figure 3C). Thus, FS patients tend to exhibit a greater abundance of immune cells in the shoulder capsule compared to normal.

Furthermore, we compared the relative levels of marker genes for each immune cell type between the two groups and found that the FS group exhibited higher levels of macrophages, M1 macrophages, basophils, CD4+ memory T cells, Th2 cells, Tregs, pDCs, B cells, memory B cells, naive B cells, and pro B cells compared to the control group (Figure 3C).

**The immune cell traits positively associated with FS are highly correlated with FS pathological features**

**Figure 4:**
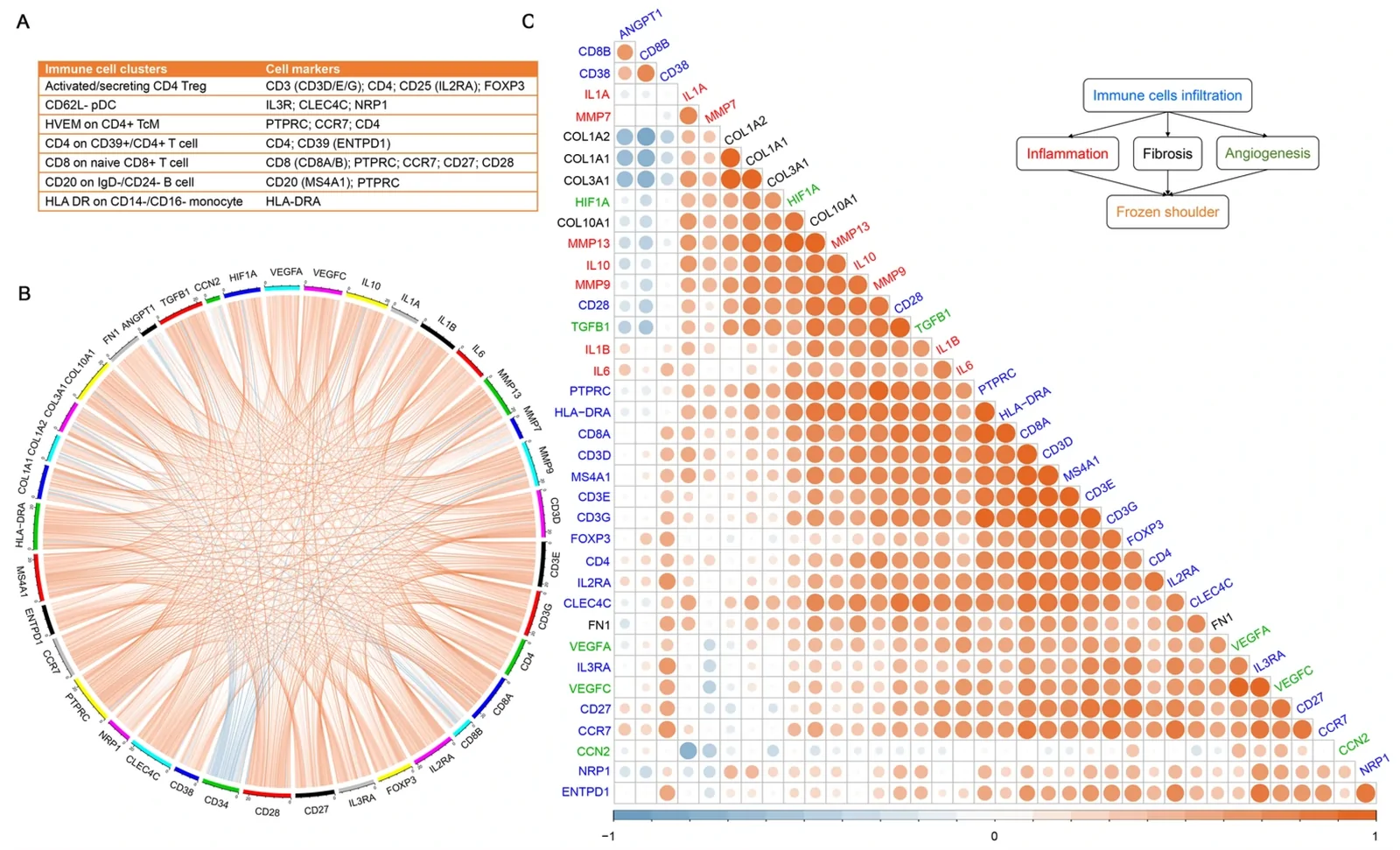
Biological characteristics and tissue expression patterns of marker genes associated with risk immune cell traits in FS. (**A**) Marker genes corresponding to the seven risk-associated immune cell traits identified by MR analysis. (**B**,**C**) Correlation networks between immune cell marker genes and inflammation-related, fibrosis-related, and angiogenesis-related genes. Immune cell marker genes are shown in blue, inflammation-related genes in red, fibrosis-related genes in black, and angiogenesis-related genes in green. Connections between nodes indicate significant correlations. Red lines denote positive correlations, whereas blue lines denote negative correlations; line thickness reflects the strength of the correlation. Tissue expression network of marker genes. Organ names are labeled directly within the figure. Connections between tissues represent significant correlations in gene expression patterns. The size and color intensity of circles indicate the magnitude of the correlation coefficient.

The immune traits that are positively correlated with the outcome of FS may have a greater impact on the progression of the disease. To confirm the roles of these seven risky immune traits in the pathological features of FS, we extracted the gene markers of these immune cells (Figure 4A) from the RNA-seq results and conducted correlation analyses with the markers of inflammation, vascularization, and fibrosis (Figure 4B,C). We found positive correlations between most of these genes, and especially that most of the immune cell markers (displayed in blue font) were positively associated with inflammation (displayed in red font), fibrosis (displayed in black font), or angiogenesis (displayed in green font) (Supplementary File S8). Tissue expression analysis demonstrated strong positive correlations among musculoskeletal and immune-related tissues, whereas only a limited number of negative correlations were observed. Thus, these findings suggest that the identified marker genes may participate in coordinated biological processes across tissues relevant to inflammatory, fibrous and angiogenetic pathology in FS progression.

**RNA-seq data verified five risky immune cell traits enriched in FS capsular tissues**

**Figure 5:**
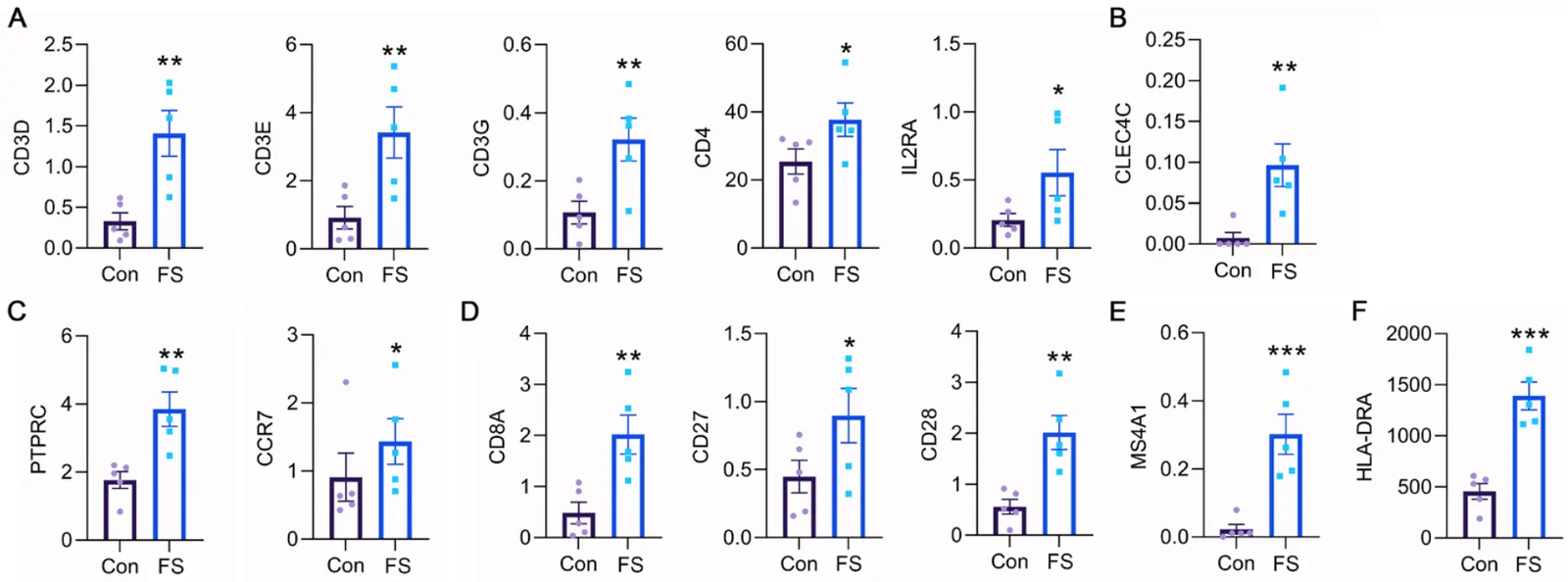
Differences in the expression of risky immune cell marker genes between the control and FS groups. (**A**) Activated/secreting CD4+ regulatory T cells. (**B**) Plasmacytoid dendritic cells (pDCs). (**C**) HVEM on CD4+ central memory T cells. (**D**) Naïve CD8+ T cells. (**E**) IgD−/CD24− B cells. (**F**) HLA-DR on CD14−/CD16− monocytes. (*: *p* < 0.05; **: *p* < 0.01; ***: *p* < 0.001).

To validate the seven risk-associated immune cell traits, including HVEM on Central Memory CD4+ T cell, CD20 on lgD− CD24− B cell, CD4 on CD39+ CD4+ T cell, CD62L-plasmacytoid Dendritic Cell, Activated and secreting CD4 regulatory T cell Absolute Count, HLA DR on CD14−CD16− monocyte and CD8 on naive CD8+ T cell, that were identified by MR analysis, marker genes corresponding to these traits were collected from the CellMarker database. Marker genes were available for six of the seven immune cell traits, resulting in a total of 13 marker genes for downstream analyses. Differential expression analysis demonstrated that marker genes corresponding to five immune cell traits were significantly enriched in FS capsular tissues compared with controls (Figure 5). The marker of activated Treg, CD25 (IL2RA), was highly expressed in the FS group. Together with CD3D/E/G and CD4, it verified the differences in activated/secreting CD4+ Treg in the FS (Figure 5A). The defining marker of the pDC lineage, CD123 (IL3R), showed no difference between the two groups, and only CLEC4C was increased in the FS group (Figure 5B). The gene markers of HVEM on CD4+ TcM, CD8 on naïve CD8+ T cells, CD20 on IgD−/CD24− B cells, and HLA DR on CD14−/CD16− monocytes were all increased in the FS group (Figure 5C–F). CD39 (ENTPD1) was not differentially expressed between the control and FS groups. This verified that there could be five cell traits participating in the process of FS.

**Causal effect of GCs on immune cell traits**

**Figure 6:**
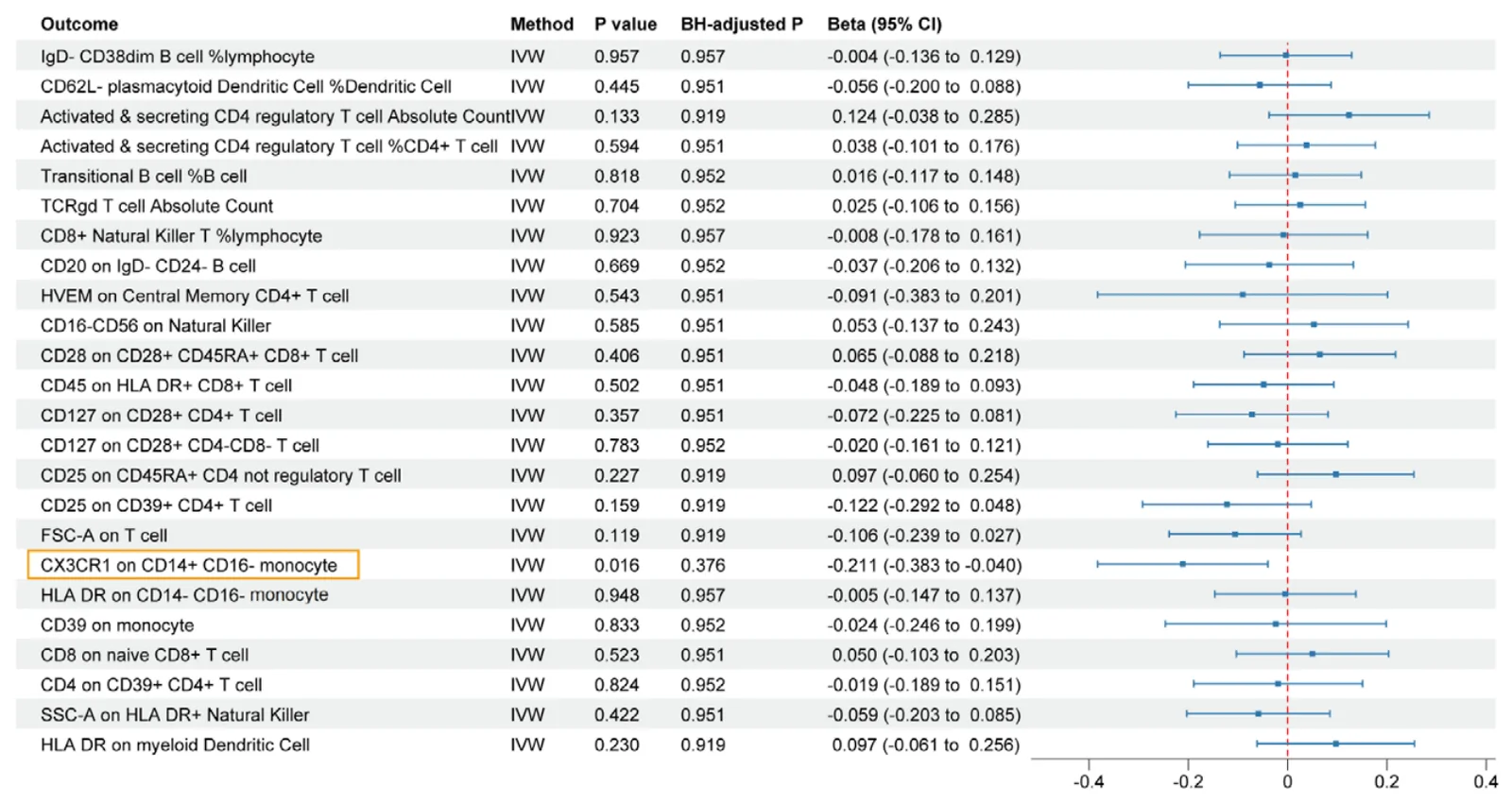
Forest plot for the causal relationship between GCs and 24 immune cell traits above from IVW.

As GCs have been widely used for treating FS in clinical practice, we further performed MR analyses to investigate the potential causal relationships among GC exposure, FS risk, and the identified immune cell traits. However, there was no correlation between GCs and FS (Supplementary Files S9 and S10). In addition, we also investigated the potential effect of GCs on these 24 immune cell traits through MR analysis. The results revealed a nominal inverse association between genetically predicted GC use and CX3CR1 on CD14+CD16− monocytes, which plays a protective role against FS (Figure 6). No causality was demonstrated for other immune cell traits (Figure 5, Supplementary Files S11 and S12). The beta values of the other four analysis methods are all in the same direction (Supplementary File S13). Leave-one-out analysis revealed that none of the SNPs could individually affect the MR analysis result (Supplementary File S14). The MR-Egger intercept test, MR-PRESSO test, Cochran’s Q test and Rucker’s Q test did not reveal any pleiotropy, heterogeneity, or outliers (Table 2).

In addition, a reverse MR analysis was also performed. The CX3CR1 on CD14+ CD16− monocyte was analyzed as an exposure with GCs as the outcome. The results of MR analysis showed no reverse causation (Supplementary File S15). Therefore, the use of GCs may not contribute to the recovery of FS but could potentially worsen the protective immune environment.

It should be noted that the absence of a significant MR association does not necessarily exclude potential therapeutic effects of GCs in specific clinical settings. Rather, our findings suggest that genetically predicted GC exposure is unlikely to exert a major direct effect on overall FS susceptibility. Given the complexity of GC pharmacology and disease-stage-specific treatment responses, further experimental and clinical studies are needed to clarify the relationship between GC therapy and FS outcomes.

## 4. Discussion

The recruitment and infiltration of immune cells may constitute a significant contributor to the worsening and chronicity of FS [1]. However, the precise roles of specific subtypes of immune cells in FS syndrome have yet to be elucidated. Compared with the previously published two-sample MR analysis by Luo et al. [15], the present study adopted a three-step framework that integrates genetic causality, tissue-level validation, and therapeutic evaluation. While both investigations exploited the same publicly available GWAS summary statistics for 731 immune cell traits, our FS outcome sample was markedly larger (627,998 vs. 451,099 individuals) and IV selection was more stringent (*p* < 5 × 10^−6^ vs. 1 × 10^−5^). We further strengthened instrument reliability by removing palindromic SNPs with ambiguous strands and by calculating F-statistics to exclude weak instruments. In addition to the IVW estimator and conventional sensitivity tests (MR-Egger, weighted median, MR-PRESSO), we applied simple/weighted mode estimators, leave-one-out analysis, and bidirectional MR to rule out reverse causation.

Utilizing RNA-seq [39], we quantified essential gene expression patterns associated with immune cells and utilized Xcell tools to identify 11 elevated immune cell subtypes in AC. To get more detailed differentiation of cell subtypes, we obtained summarized statistical information on 731 immune cell traits [14], and identified 7 traits as risk factors for FS, while 17 traits had protective effects. Using RNA-seq, we observed the upregulated immune cell subtypes in FS. However, we cannot determine whether these subtypes promote or protect the FS process. By combining with the MR results, we can further assess their specific roles in FS.

Combining MR and RNA-seq, the data revealed four T cell traits associated with an elevated risk of FS, including HVEM on Central Memory CD4+ T cells, Activated and secreting CD4 Treg Absolute Count, CD4 on CD4+ CD39+ cells, and CD8 on naive CD8+ T cells, along with Th2 cells that increased in FS with unknown roles. CD4+ T cells have the ability to differentiate into distinct subpopulations, such as Th1, Th2 and Tregs. Previous studies have established a correlation between Th cells and tissue fibrosis [40]. Th2 was reported to exacerbate cystic fibrosis induced by pseudomonas aeruginosa infection [41]. Furthermore, Tregs have been linked to fibrosis in cardiopulmonary and lymphoid tissues [42]. Consequently, the aberrant proliferation of certain T cells in individuals with FS may play a role in the advancement of the condition through the induction of fibrosis.

For B cells, we identified increased memory B cells, naive B cells, and pro B cells in FS with unknown roles, and CD20 on lgD- CD24- B cells as a potential risk of FS. While CD20 is universally expressed on all B cells, its presence on lgD- CD24- B cells may not confer significant specificity [43]. Additionally, MR analysis also highlighted a HLA DR on CD14− CD16− monocyte that positively related to FS. The precise mechanism underlying the role of monocytes in FS remains unclear, but their association with inflammation has been previously documented [44]. In line with the RNA-seq results, MR found that CD62L-pDC increases the risk of FS, which may be related to its roles in inflammatory injury through antigen presentation and immunomodulation [45].

Interestingly, more immune cell traits with protective characteristics were found compared to those with pathogenic characteristics for FS from MR analysis. Notably, distinct cell markers of the same immune cells can have opposing effects on FS. CD25, also known as the IL-2 receptor alpha chain, is typically expressed on activated T lymphocytes and Tregs [46]. In individuals with autoimmune diseases, like juvenile idiopathic arthritis, there is a notable decrease in CD25 expression on CD4+ T cells, which may disrupt the balance between pro-inflammatory T cells and Tregs, leading to a breakdown in self-tolerance [47]. Consequently, the presence of CD25 on CD39+ CD4+ T cells could potentially influence FS by modulating immune homeostasis. Similarly, protective CD25 on CD14RA+ CD4 not Tregs and activated and secreting CD4 Tregs may have the same effects. Tregs, as a subset of immunosuppressive T cells, play a crucial role in mitigating the risk of autoimmune disorders and excessive inflammatory responses [48]. In FS, moderate Tregs may maintain shoulder tissue homeostasis by modulating inflammatory responses [1]. However, excessive proliferation of Tregs may exacerbate the risk of FS by promoting tissue fibrosis as described above [42,49]. Therefore, the maintenance of Treg functionality at optimal levels is essential for resistance to FS.

In the present MR study, it was observed that two CD8+ T cell subsets, except for inactivated naive CD8+ cells, exhibited a protective effect on FS. CD8+ T cells, also known as memory T cells, function by recognizing specific antigens to eliminate cancerous cells or clear pathogens and damaged cells [50]. Mature CD8+ T cells present a rapid responsiveness to stimuli [51], contributing to their protective function over naive CD8+ T cells. However, CD8+ T cells in the initial stages of disease can still maintain the intra-articular environment by eliminating pathogens or diseased cells [52]. Furthermore, CD8+ T cells have the capability to mitigate autoimmune disease through the inhibition of autoreactive immune cells [53].

LgD-CD38 dim B cells and Transitional B cells were also demonstrated to be protective in FS. CD38, as a B cell surface receptor, is a key regulatory molecule in the maturation of B cells. It has been demonstrated that CD38− B cells promote autoimmune responses and exacerbate autoimmune diseases such as autoimmune diabetes and rheumatoid arthritis, whereas activation of CD38 promotes IL-10 expression by regulatory B cells, thereby suppressing excessive inflammation [54]. These findings suggest a potential protective mechanism for LgD-CD38 dim B cells and Transitional B cells in FS.

The protective effect of CX3CR1 on CD14+CD16− monocytes contrasted with the opposite effect observed for HLA-DR on CD14−CD16− monocytes. HLA-DR is involved in antigen presentation, whereas CX3CR1 interacts with CX3CL1 and has been implicated in limiting inflammatory responses [55,56]. Therefore, the protective association of CX3CR1 may reflect the anti-inflammatory function of this monocyte subset. Similarly, mDCs showed effects opposite to pDCs, suggesting distinct roles of dendritic-cell subsets in FS. Although their functions vary across inflammatory conditions, mDCs may influence immune regulation and tissue homeostasis [57].

Three NK-cell traits in this study, including SSC-A on HLA-DR+ NK cells, CD16−CD56 on NK cells, and CD8+ NK T %lymphocyte, were associated with reduced FS risk. NK cells regulate immune responses through cytokine production, elimination of activated immune cells, and modulation of macrophage activity. The CD16−CD56 phenotype corresponds to the CD56brightCD16− NK-cell subset, which is involved in immune regulation rather than antibody-dependent cytotoxicity [58,59]. In chronic inflammatory and fibrotic diseases, NK cells may help maintain immune homeostasis and limit excessive tissue remodeling. These findings suggest that specific NK-cell phenotypes may have protective roles in FS, although further mechanistic studies are required.

In clinical practice, intra-articular corticosteroid injections are commonly used during the early painful stage of FS, when synovial inflammation is considered a predominant feature. Regimens such as triamcinolone acetonide (20–40 mg) or methylprednisolone acetate (40 mg) [60], as well as short courses of oral prednisolone, may provide short-term symptom relief; however, their long-term efficacy remains inconsistent [61]. Typical interventions for early FS include physical therapy, nonsteroidal anti-inflammatory drugs, and GCs. GCs have shown effectiveness in improving the range of motion and reducing pain in the short term, making them a commonly used pharmacological treatment for FS [62,63,64]. Therefore, early use of appropriate doses of GCs can effectively relieve the symptoms of FS and improve patients’ quality of life, but the long-term effects and the final outcome of FS remain unclear. Local complications of intra-articular injections include post-injection pain, skin depigmentation, and, rarely, infection. Systemic corticosteroid exposure may cause hyperglycemia, hypertension, weight gain, osteoporosis, adrenal suppression, and increased susceptibility to infection, particularly with repeated or prolonged use.

Although GCs are widely used in FS management due to their anti-inflammatory effects, our MR analyses did not identify a direct causal association between genetically predicted GC exposure and FS risk. Interestingly, genetically predicted GC exposure showed a negative causal association with CX3CR1 expression on CD14+CD16− monocytes, a protective immune cell trait associated with reduced FS risk. CX3CR1 contributes to monocyte trafficking, immune surveillance, tissue repair, and resolution of inflammation, and CX3CR1-positive monocytes have been implicated in maintaining immune homeostasis and limiting excessive inflammatory and fibrotic responses. These findings suggest that GC therapy may exert a potential trade-off by alleviating inflammation while simultaneously suppressing certain protective immune mechanisms. Although GCs remain effective for short-term symptom control in FS, this finding suggests that their immunological effects may be more complex than previously appreciated and warrant further investigation. Consequently, despite the short-term benefits of GCs in alleviating symptoms of FS, their adverse effects afterwards may involve the CX3CR1 receptor on CD14+CD16− monocytes. Further mechanistic and clinical studies are required to determine how GC-induced immune alterations influence disease progression in FS.

This study represents the most comprehensive analysis to date of the causal relationship between 731 immune cell traits, FS and GCs. An important finding of this study is the apparent “double-edged” role of immune cell genetic signatures in FS. Rather than indicating a uniformly pathogenic immune response, our MR analyses identified both risk-associated (7 traits) and protective (17 traits) immune cell traits, suggesting a complex balance between pro-inflammatory/pro-fibrotic mechanisms and compensatory immune regulation. This finding is consistent with the context-dependent functions of immune subsets in chronic inflammatory and fibrotic disorders, where different populations may either promote or restrain pathological tissue remodeling [65,66]. Therefore, immune-targeted therapies for FS should consider both pathogenic and protective immune mechanisms rather than broadly suppressing immune activity.

Nevertheless, it is important to acknowledge the limitations of our MR study. Relying solely on GWAS data from European populations restricts the generalizability of our findings. The Chinese clinical samples were used only for RNA-seq validation rather than SNP-level MR analysis; therefore, ancestry-related differences in allele frequencies and linkage disequilibrium patterns may limit the generalizability of our findings to non-European populations. In addition, although RNA-seq provided independent transcriptomic support for the MR results, further validation at the mRNA and protein levels is still required to confirm the biological functions of the identified immune cell traits and their marker genes. It is essential to corroborate certain findings and inferences with biological evidence to strengthen the validity of this study. Although experimental validation remains indispensable for elucidating biological mechanisms, MR complements conventional observational studies by strengthening causal inference while reducing confounding and reverse causation through the use of genetic variants. Accordingly, our findings should be regarded as genetically informed evidence that generates mechanistic hypotheses rather than definitive biological proof.

## 5. Conclusions

**Figure 7:**
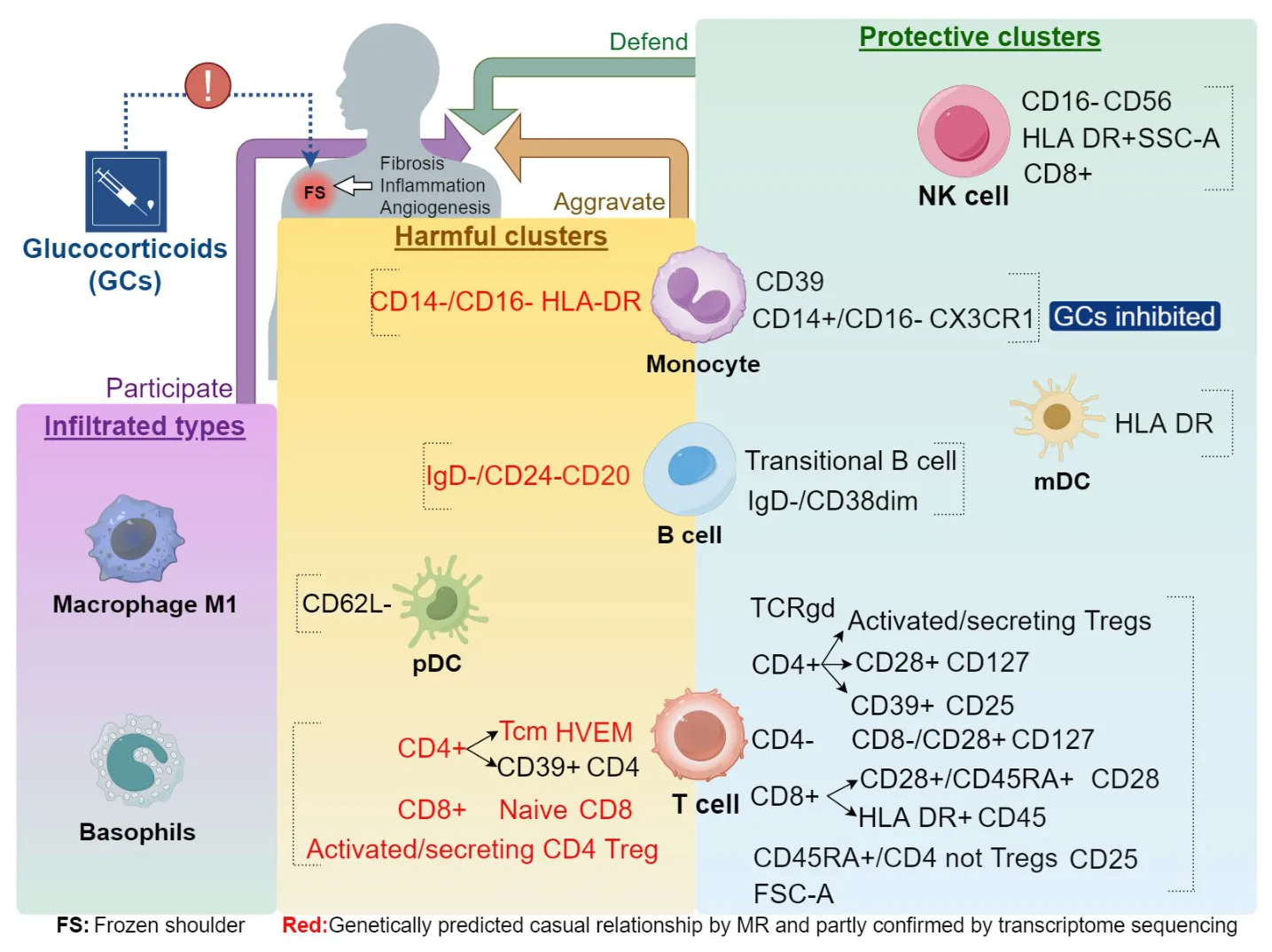
Overview of the causal relationship among immune cell traits, FS, and GCs. Seven immune cell traits were identified as risk factors for FS pathology (inflammation, fibrosis, angiogenesis), five of which were confirmed to be upregulated through the RNA-seq analysis (depicted in red font), along with 17 immune cell traits protective against FS. RNA-seq also identified an M1-macrophage and basophils that are significantly upregulated in FS. GCs only have a negative causal association with CX3CR1 on CD14+CD16− monocytes.

Using MR analysis, we identified seven immune cell traits as risk factors for FS pathology, five of which were confirmed to be upregulated through the RNA-seq analysis (depicted in red font in Figure 7), along with 17 immune cell traits protective against FS. In addition to the overlap with the MR results, RNA-seq also identified the upregulated M1-macrophage and basophils in FS, and their specific roles remain to be further verified. Furthermore, the treatment of FS using GCs should be thoroughly considered on a case-by-case basis because, genetically, instead of contributing to the regression of FS, it may disrupt the immune microenvironment and have a detrimental effect. Based on the mutual complementarity and validation of MR and RNA-seq, we identified the double-edged effects of genetic signatures of immune cells on FS, providing novel ideas for future immune cell-based precision therapeutic interventions.

## Figures and Tables

**Table 1:** Associations of genetically predicted immune cells with FS in sensitivity analyses.

Exposure	Heterogeneity		Pleiotropy		MR-PRESSO
	MR Egger	IVW	MR Egger		
	Q_*p*val	Q_*p*val	Intercept	*p*val	*p*val
IgD− CD38dim B cell %lymphocyte	0.254	0.320	0.011	0.566	0.360
CD62L− plasmacytoid Dendritic Cell %Dendritic Cell	0.957	0.827	0.012	0.287	0.867
Activated and secreting CD4 regulatory T cell Absolute Count	NA	0.805	NA	NA	NA
Activated and secreting CD4 regulatory T cell %CD4+ T cell	0.406	0.572	−0.005	0.700	0.619
Transitional B cell %B cell	0.444	0.554	0.008	0.600	0.601
TCRgd T cell Absolute Count	0.443	0.516	−0.017	0.518	0.557
CD20 on IgD− CD24− B cell	0.752	0.346	0.038	0.240	0.464
HVEM on Central Memory CD4+ T cell	NA	0.680	NA	NA	NA
CD16-CD56 on Natural Killer	0.606	0.683	0.004	0.750	0.696
CD28 on CD28+ CD45RA+ CD8+ T cell	0.155	0.152	0.028	0.409	0.223
CD45 on HLA DR+ CD8+ T cell	0.546	0.611	−0.011	0.514	0.561
CD127 on CD28+ CD4+ T cell	0.460	0.503	0.008	0.456	0.528
CD127 on CD28+ CD4−CD8− T cell	0.790	0.725	−0.019	0.347	0.767
CD25 on CD45RA+ CD4 not regulatory T cell	0.437	0.563	−0.001	0.890	0.752
CD25 on CD39+ CD4+ T cell	0.127	0.185	0.008	0.733	0.227
FSC-A on T cell	NA	0.839	NA	NA	NA
CX3CR1 on CD14+ CD16− monocyte	0.061	0.099	0.005	0.839	0.143
HLA DR on CD14− CD16− monocyte	0.123	0.081	−0.019	0.229	0.088
CD39 on monocyte	0.605	0.654	−0.004	0.530	0.804
CD8 on naive CD8+ T cell	0.425	0.547	−0.003	0.833	0.624
CD4 on CD39+ CD4+ T cell	0.902	0.939	−0.003	0.782	0.927
SSC-A on HLA DR+ Natural Killer	0.590	0.677	−0.005	0.658	0.757
HLA DR on myeloid Dendritic Cell	0.184	0.128	−0.018	0.263	0.297

**Table 2:** Associations of genetically predicted medication use (GCs) with immune cells in sensitivity analyses.

	Heterogeneity		Pleiotropy		MR-PRESSO
Outcome	MR Egger	IVW	MR Egger		
	Q_*p*val	Q_*p*val	Intercept	*p*val	*p*val
CX3CR1 on CD14+ CD16− monocyte	0.043	0.048	0.025	0.470	0.061

## Data Availability

The original contributions presented in the study are included in the article or Supplementary Material. Further inquiries can be directed to the corresponding authors.

## Supplementary Materials

The following supporting information can be downloaded at: https://www.mdpi.com/article/10.3390/biomedicines14081699/s1.

## Abbreviations

The following abbreviations are used in this manuscript:

FS	frozen shoulder
RNA-seq	RNA-sequencing
MR	Mendelian randomization
SNPs	single nucleotide polymorphisms
GCs	glucocorticoids
IVs	instrumental variables
GWAS	genome-wide association studies
AC	absolute cell
MFIs	median fluorescence intensities
MP	morphological parameters
LD	linkage disequilibrium
IVW	inverse variance weighted
95% CI	95% confidence interval
TNF	tumor necrosis factor
IL	interleukin
IFN	interferon
HIF1A	hypoxia-inducible factor-1
TGFβ1	transforming growth factor beta 1
CCN2	cellular communication network factor 2
NK	natural killer
mDC	myeloid dendritic cell
pDC	plasmacytoid dendritic cell
Treg	regulatory T cell
Th	T helper
COL	collagen
FN1	fibronectin 1
