# Supplementary material for: Immune Cell Causality and Glucocorticoid Trade-Off in Frozen Shoulder: Integrative MR-RNA-Seq Analysis
Source: Biomedicines. 2026 Jul 29;14(8):1699. doi: 10.3390/biomedicines14081699 (PMC13509323; doi:10.3390/biomedicines14081699)
Supplement: Supplementary file 1 [file biomedicines-14-01699-s001.zip › Supplementary File S3.pdf]

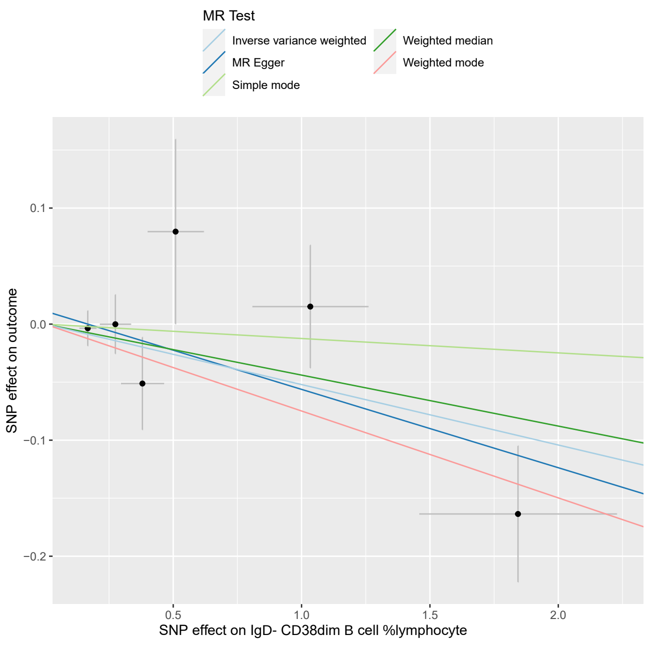
MR Test
Inverse variance weighted
MR Egger
Simple mode
Weighted median
Weighted mode
SNP effect on outcome
0.1
0.0
−0.1
−0.2
0.5
1.0
1.5
2.0
SNP effect on IgD- CD38dim B cell %lymphocyte

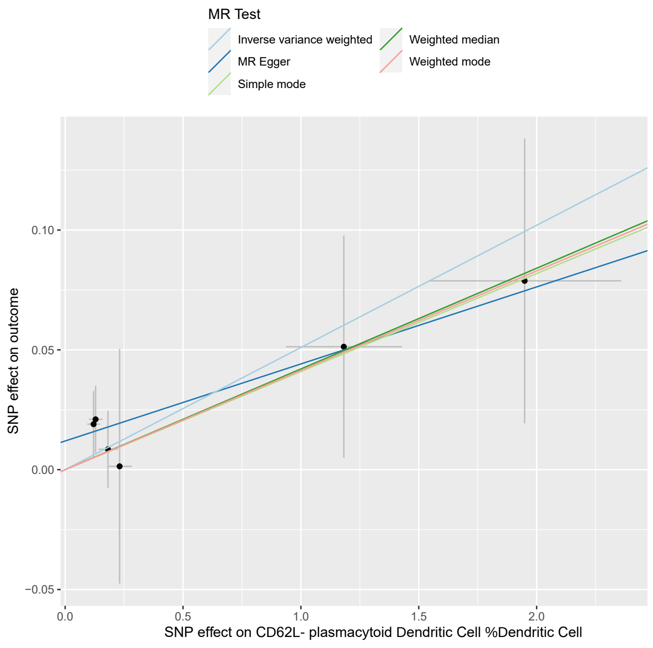
MR Test
Inverse variance weighted
MR Egger
Simple mode
Weighted median
Weighted mode
SNP effect on outcome
0.10
0.05
0.00
−0.05
0.0
0.5
1.0
1.5
2.0
SNP effect on CD62L- plasmacytoid Dendritic Cell %Dendritic Cell

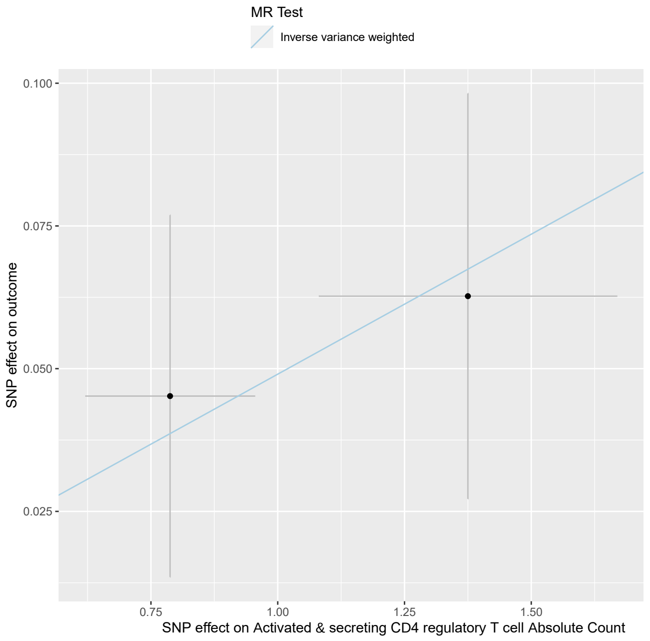
MR Test
Inverse variance weighted
SNP effect on outcome
0.100
0.075
0.050
0.025
0.75
1.00
1.25
1.50
SNP effect on Activated & secreting CD4 regulatory T cell Absolute Count

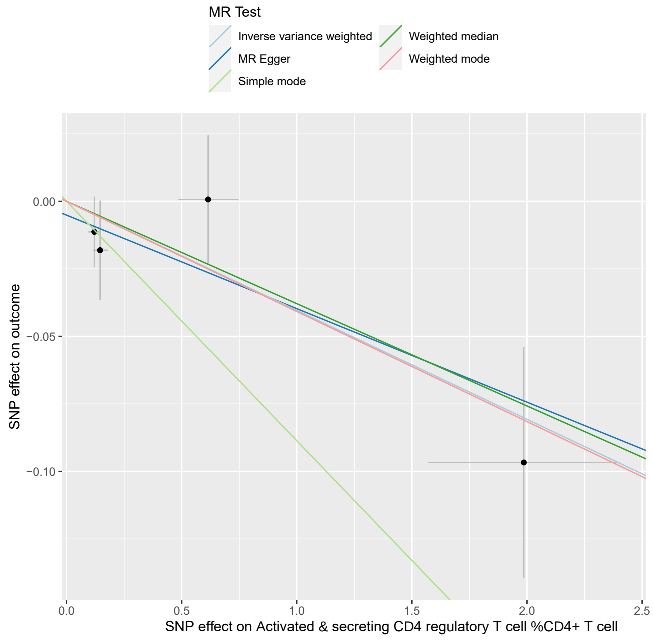
MR Test
Inverse variance weighted
MR Egger
Simple mode
Weighted median
Weighted mode
SNP effect on outcome
0.00
−0.05
−0.10
0.0
0.5
1.0
1.5
2.0
2.5
SNP effect on Activated & secreting CD4 regulatory T cell %CD4+ T cell

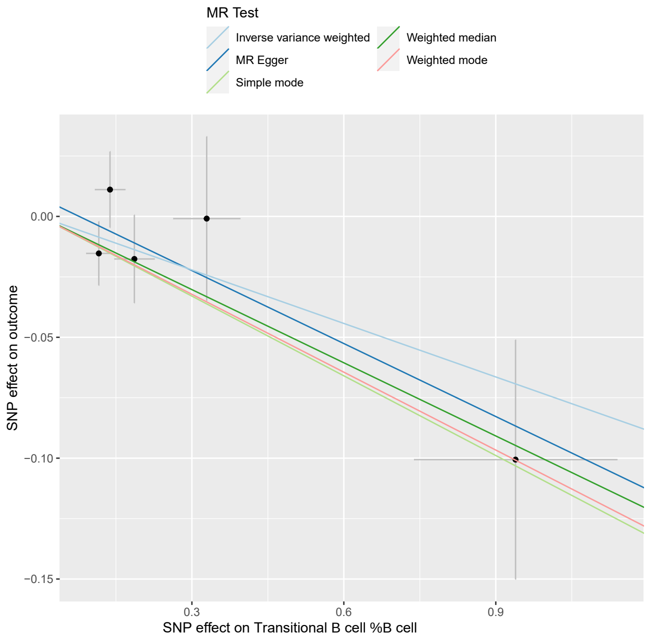
MR Test
Inverse variance weighted
MR Egger
Simple mode
Weighted median
Weighted mode
SNP effect on outcome
0.00
−0.05
−0.10
−0.15
0.3
0.6
0.9
SNP effect on Transitional B cell %B cell

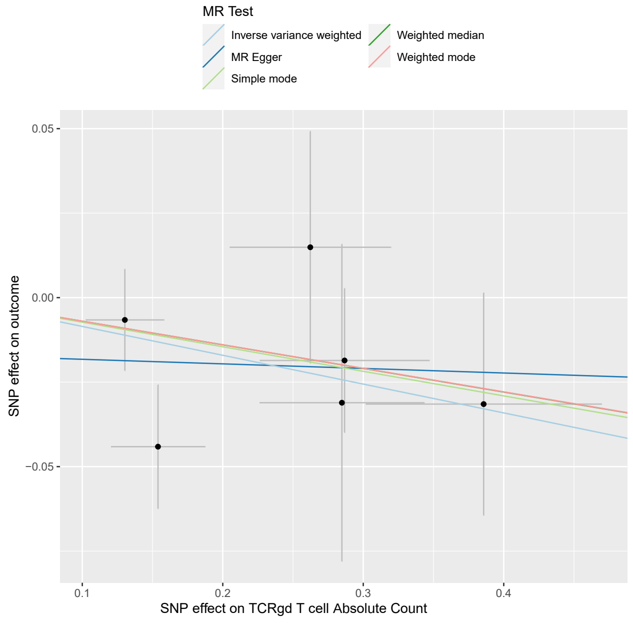
MR Test
Inverse variance weighted
MR Egger
Simple mode
Weighted median
Weighted mode
SNP effect on outcome
0.05
0.00
−0.05
0.1
0.2
0.3
0.4
SNP effect on TCRgd T cell Absolute Count

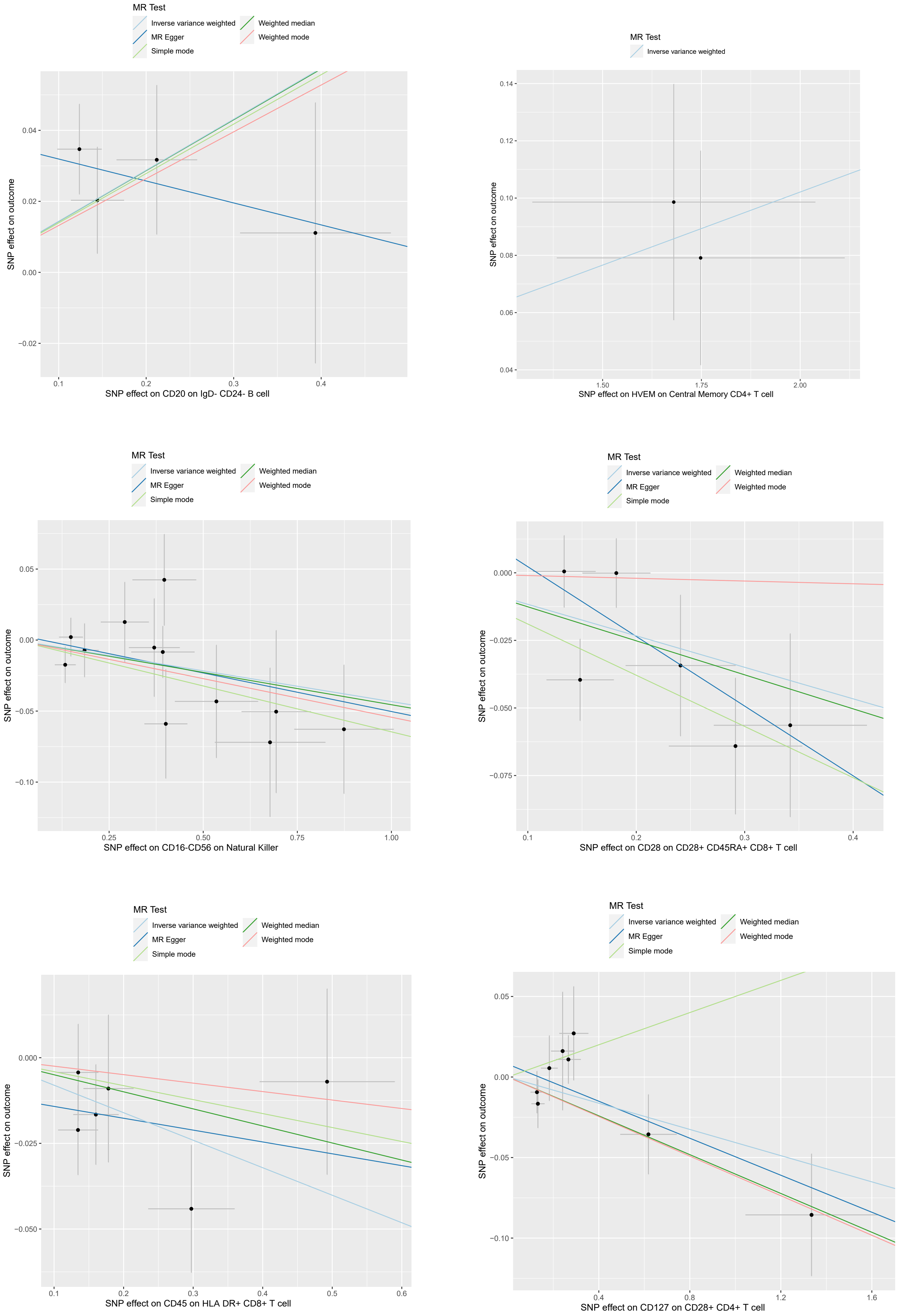
MR Test
Inverse variance weighted
MR Egger
Simple mode
Weighted median
Weighted mode
SNP effect on outcome
0.04
0.02
0.00
−0.02
0.1
0.2
0.3
0.4
SNP effect on CD20 on IgD- CD24- B cell
MR Test
Inverse variance weighted
SNP effect on outcome
0.14
0.12
0.10
0.08
0.06
0.04
1.50
1.75
2.00
SNP effect on HVEM on Central Memory CD4+ T cell
MR Test
Inverse variance weighted
MR Egger
Simple mode
Weighted median
Weighted mode
SNP effect on outcome
0.05
0.00
−0.05
−0.10
0.25
0.50
0.75
1.00
SNP effect on CD16-CD56 on Natural Killer
MR Test
Inverse variance weighted
MR Egger
Simple mode
Weighted median
Weighted mode
SNP effect on outcome
0.000
−0.025
−0.050
−0.075
0.1
0.2
0.3
0.4
SNP effect on CD28 on CD28+ CD45RA+ CD8+ T cell
MR Test
Inverse variance weighted
MR Egger
Simple mode
Weighted median
Weighted mode
SNP effect on outcome
0.000
−0.025
−0.050
0.1
0.2
0.3
0.4
0.5
0.6
SNP effect on CD45 on HLA DR+ CD8+ T cell
MR Test
Inverse variance weighted
MR Egger
Simple mode
Weighted median
Weighted mode
SNP effect on outcome
0.05
0.00
−0.05
−0.10
0.4
0.8
1.2
1.6
SNP effect on CD127 on CD28+ CD4+ T cell

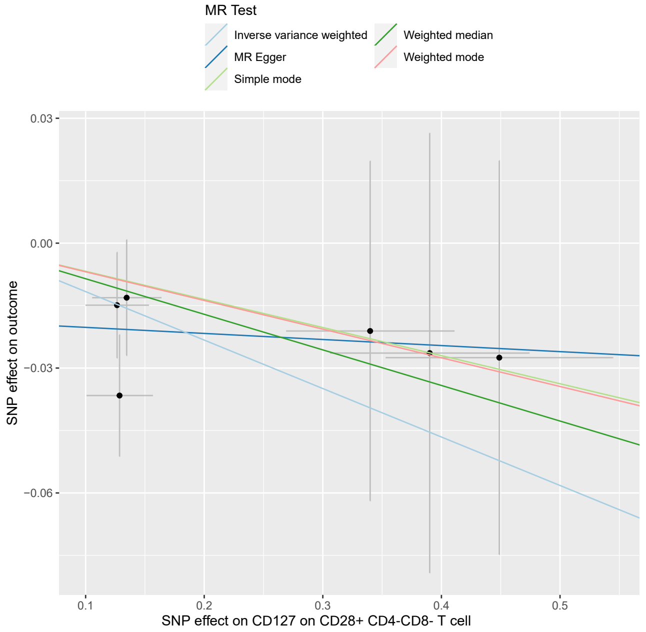
MR Test
Inverse variance weighted
MR Egger
Simple mode
Weighted median
Weighted mode
0.03
0.00
−0.03
−0.06
0.1
0.2
0.3
0.4
0.5
SNP effect on outcome
SNP effect on CD127 on CD28+ CD4-CD8- T cell

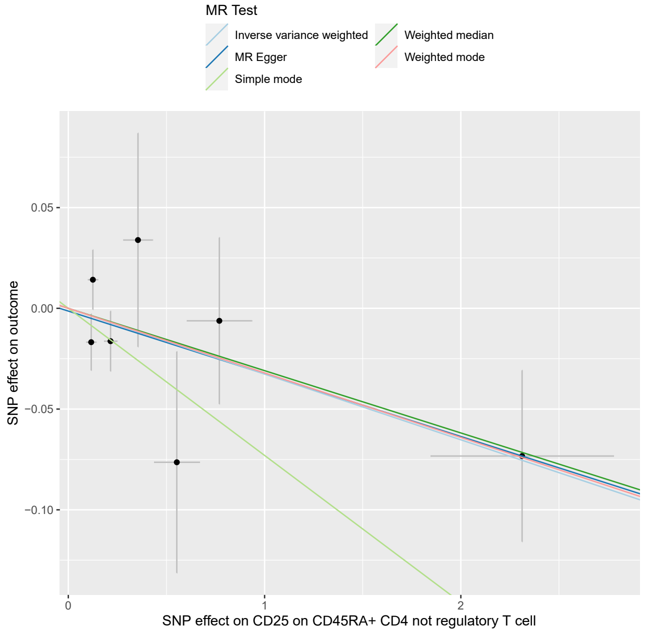
MR Test
Inverse variance weighted
MR Egger
Simple mode
Weighted median
Weighted mode
0.05
0.00
−0.05
−0.10
0
1
2
SNP effect on outcome
SNP effect on CD25 on CD45RA+ CD4 not regulatory T cell

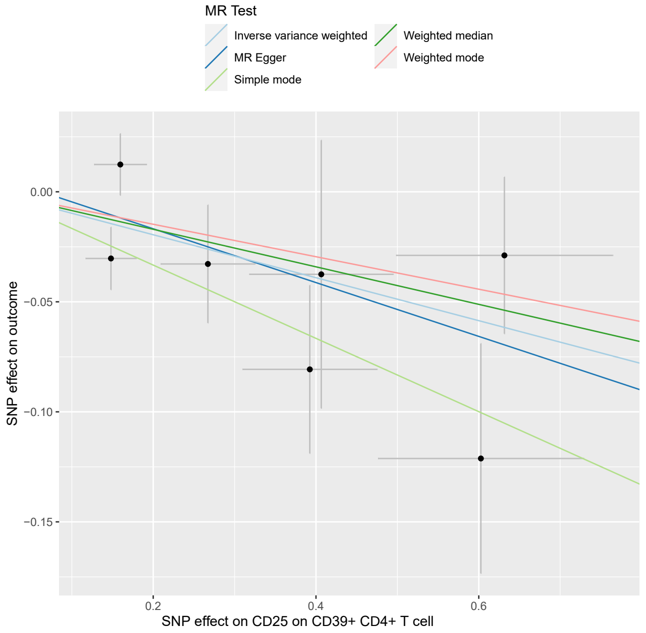
MR Test
Inverse variance weighted
MR Egger
Simple mode
Weighted median
Weighted mode
0.00
−0.05
−0.10
−0.15
0.2
0.4
0.6
SNP effect on outcome
SNP effect on CD25 on CD39+ CD4+ T cell

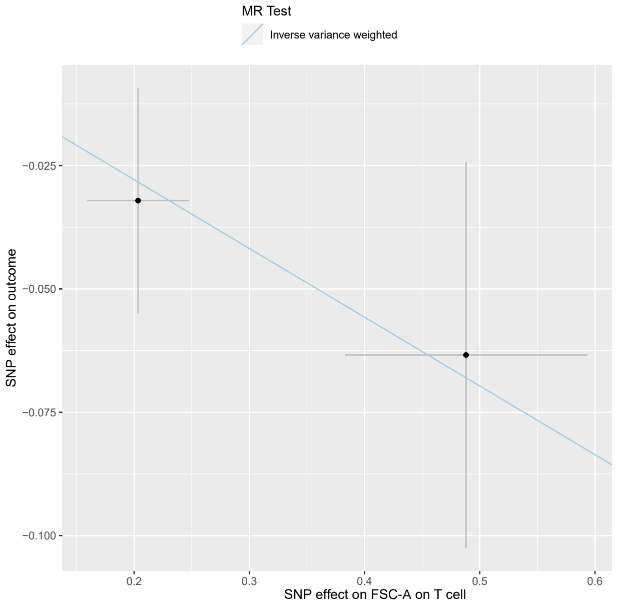
MR Test
Inverse variance weighted
−0.025
−0.050
−0.075
−0.100
0.2
0.3
0.4
0.5
0.6
SNP effect on outcome
SNP effect on FSC-A on T cell

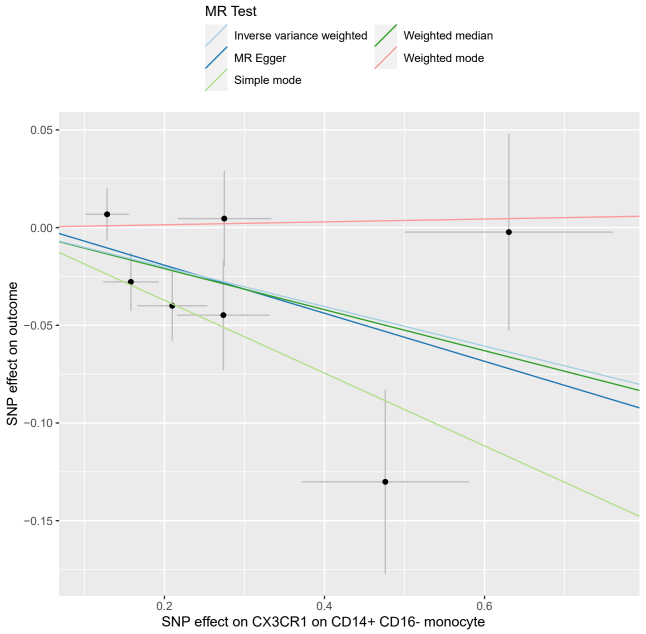
MR Test
Inverse variance weighted
MR Egger
Simple mode
Weighted median
Weighted mode
0.05
0.00
−0.05
−0.10
−0.15
0.2
0.4
0.6
SNP effect on outcome
SNP effect on CX3CR1 on CD14+ CD16- monocyte

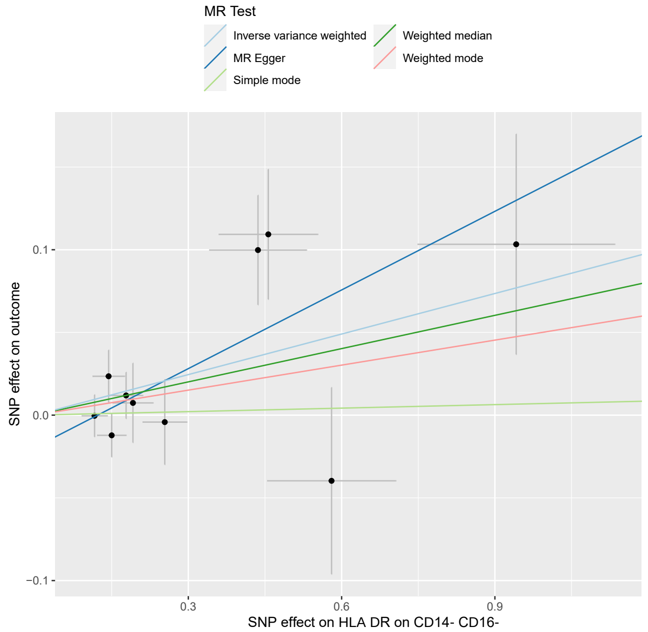
MR Test
Inverse variance weighted
MR Egger
Simple mode
Weighted median
Weighted mode
0.1
0.0
−0.1
0.3
0.6
0.9
SNP effect on outcome
SNP effect on HLA DR on CD14- CD16-

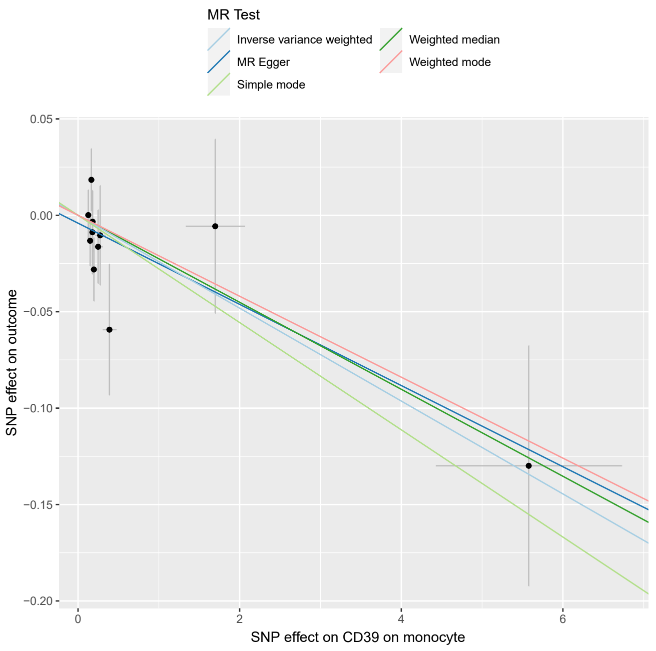
MR Test
Inverse variance weighted
MR Egger
Simple mode
Weighted median
Weighted mode
0.05
0.00
−0.05
−0.10
−0.15
−0.20
0
2
4
6
SNP effect on outcome
SNP effect on CD39 on monocyte

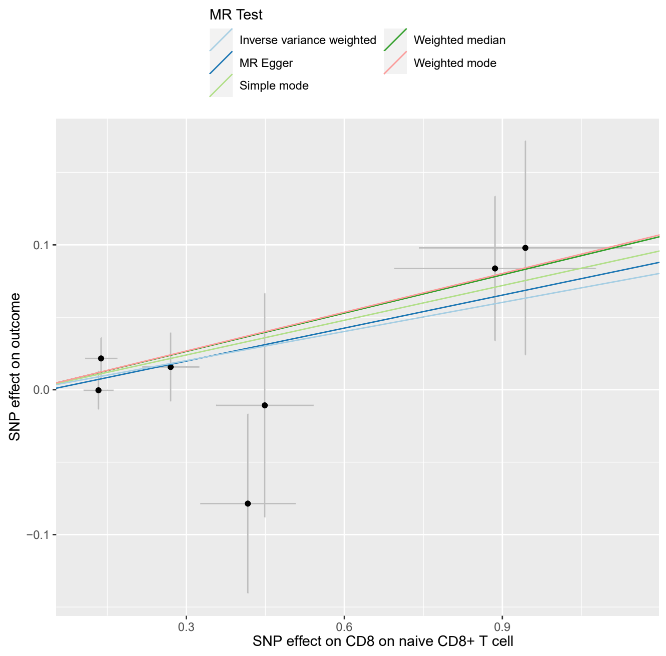
MR Test
Inverse variance weighted
MR Egger
Simple mode
Weighted median
Weighted mode
0.1
0.0
−0.1
0.3
0.6
0.9
SNP effect on outcome
SNP effect on CD8 on naive CD8+ T cell

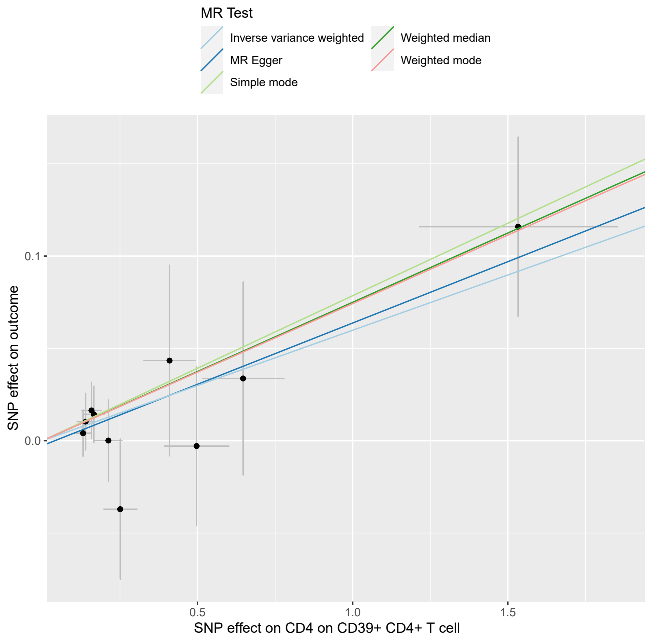
MR Test
Inverse variance weighted
MR Egger
Simple mode
Weighted median
Weighted mode
0.1
0.0
0.5
1.0
1.5
SNP effect on outcome
SNP effect on CD4 on CD39+ CD4+ T cell

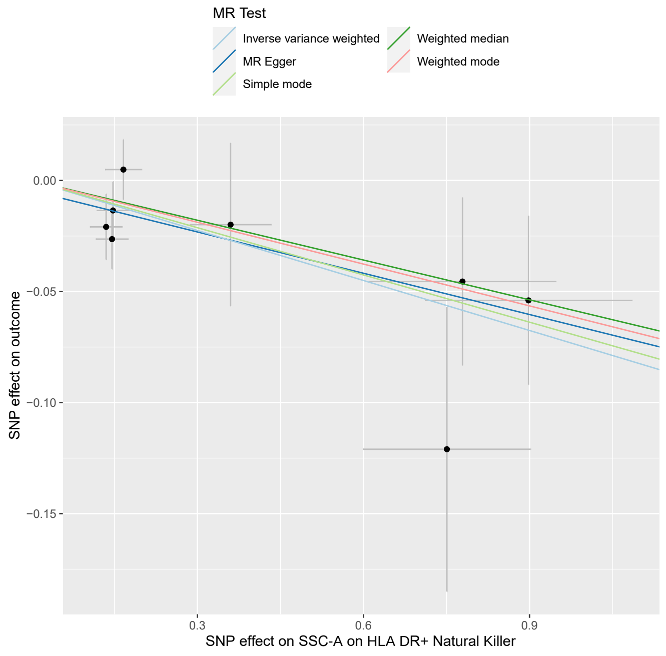
MR Test
Inverse variance weighted
MR Egger
Simple mode
Weighted median
Weighted mode
0.00
−0.05
−0.10
−0.15
0.3
0.6
0.9
SNP effect on outcome
SNP effect on SSC-A on HLA DR+ Natural Killer

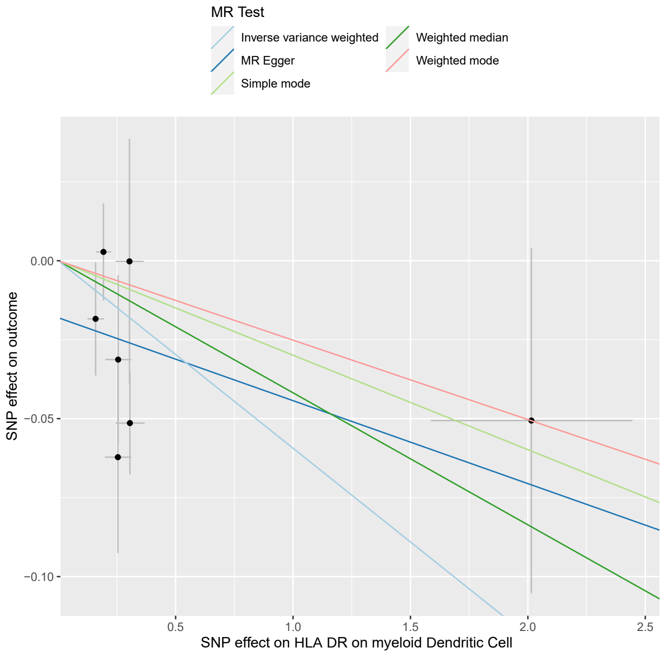
MR Test
Inverse variance weighted
MR Egger
Simple mode
Weighted median
Weighted mode
0.00
−0.05
−0.10
0.5
1.0
1.5
2.0
2.5
SNP effect on outcome
SNP effect on HLA DR on myeloid Dendritic Cell
