# Supplementary material for: Immune Cell Causality and Glucocorticoid Trade-Off in Frozen Shoulder: Integrative MR-RNA-Seq Analysis
Source: Biomedicines. 2026 Jul 29;14(8):1699. doi: 10.3390/biomedicines14081699 (PMC13509323; doi:10.3390/biomedicines14081699)
Supplement: Supplementary file 1 [file biomedicines-14-01699-s001.zip › Supplementary File S4.pdf]

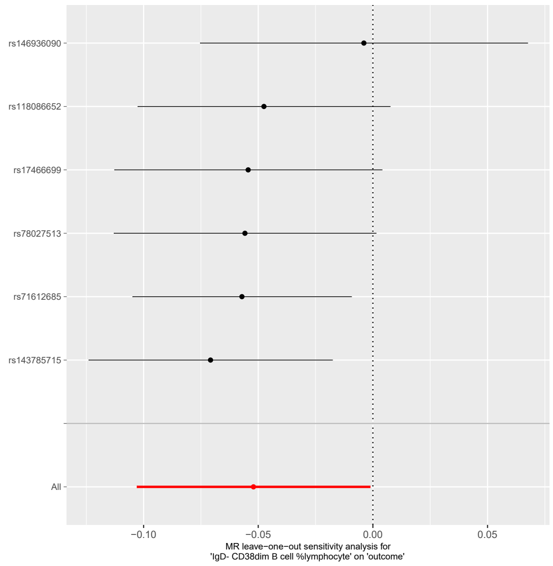
rs146936090
rs118086652
rs17466699
rs78027513
rs71612685
rs143785715
All
−0.10
−0.05
0.00
0.05
MR leave−one−out sensitivity analysis for
'IgD- CD38dim B cell %lymphocyte' on 'outcome'

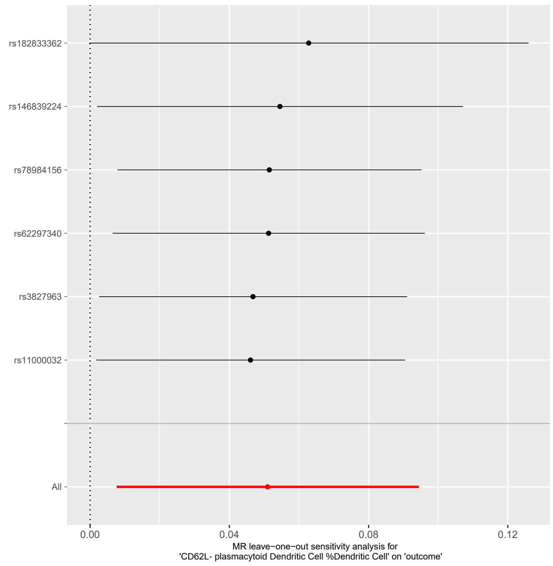
rs182833362
rs146839224
rs78984156
rs62297340
rs3827963
rs11000032
All
0.00
0.04
0.08
0.12
MR leave−one−out sensitivity analysis for
'CD62L- plasmacytoid Dendritic Cell %Dendritic Cell' on 'outcome'

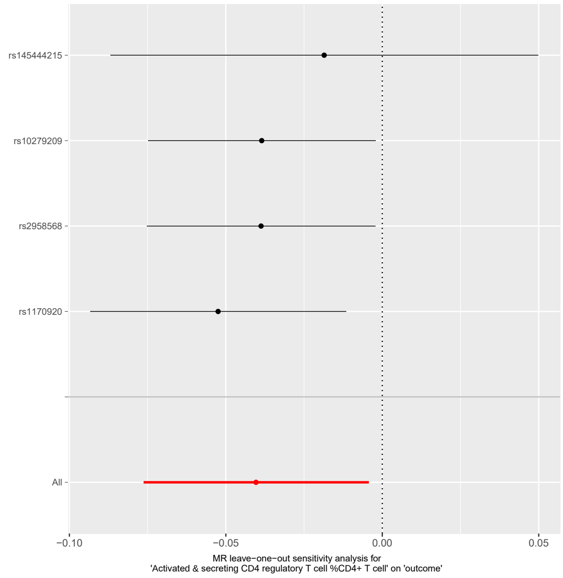
rs145444215
rs10279209
rs2958568
rs1170920
All
−0.10
−0.05
0.00
0.05
MR leave−one−out sensitivity analysis for
'Activated & secreting CD4 regulatory T cell %CD4+ T cell' on 'outcome'

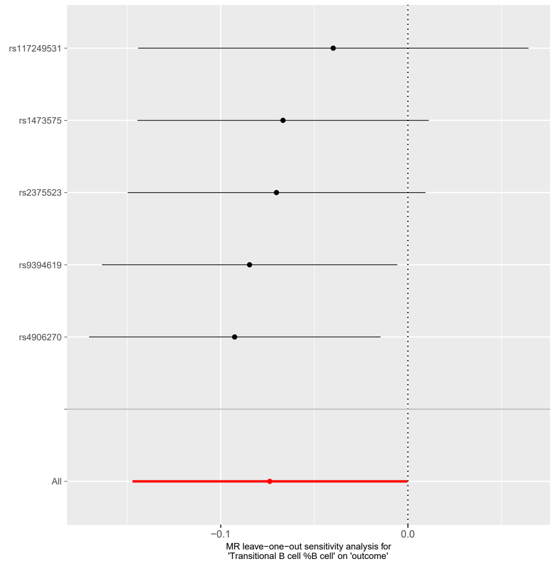
rs117249531
rs1473575
rs2375523
rs9394619
rs4906270
All
−0.1
0.0
MR leave−one−out sensitivity analysis for
'Transitional B cell %B cell' on 'outcome'

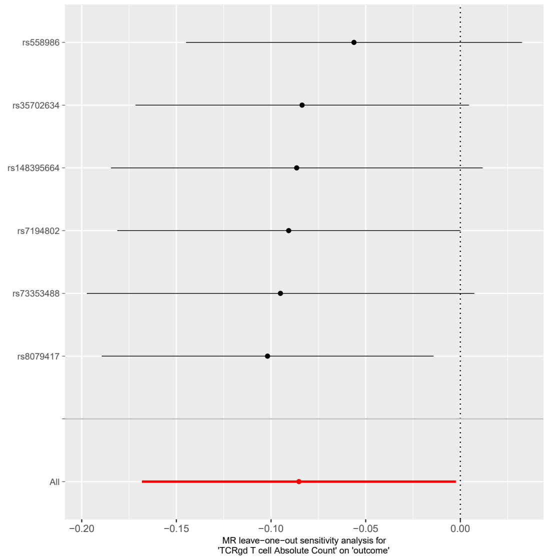
rs558986
rs35702634
rs148395664
rs7194802
rs73353488
rs8079417
All
−0.20
−0.15
−0.10
−0.05
0.00
MR leave−one−out sensitivity analysis for
'TCRgd T cell Absolute Count' on 'outcome'

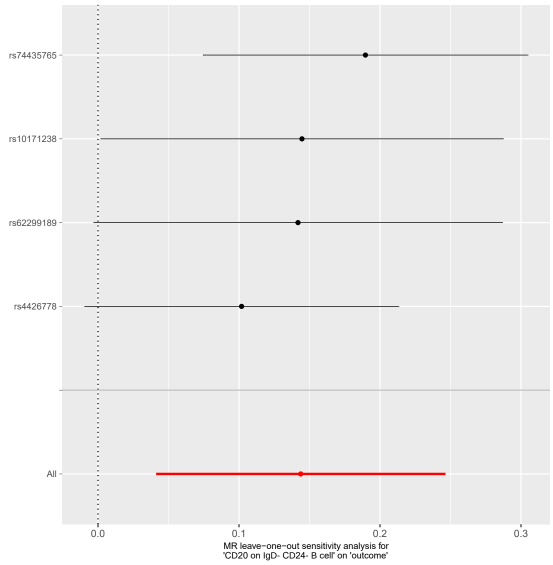
rs74435765
rs10171238
rs62299189
rs4426778
All
0.0
0.1
0.2
0.3
MR leave−one−out sensitivity analysis for
'CD20 on IgD- CD24- B cell' on 'outcome'

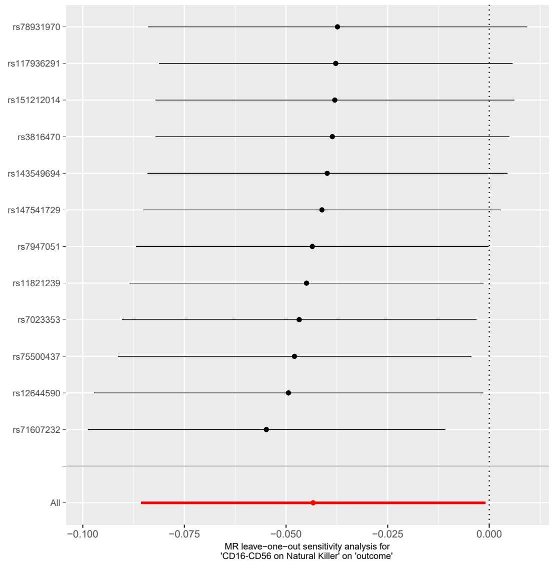
rs78931970
rs117936291
rs151212014
rs3816470
rs143549694
rs147541729
rs7947051
rs11821239
rs7023353
rs75500437
rs12644590
rs71607232
All
−0.100
−0.075
−0.050
−0.025
0.000
MR leave−one−out sensitivity analysis for
'CD16-CD56 on Natural Killer' on 'outcome'

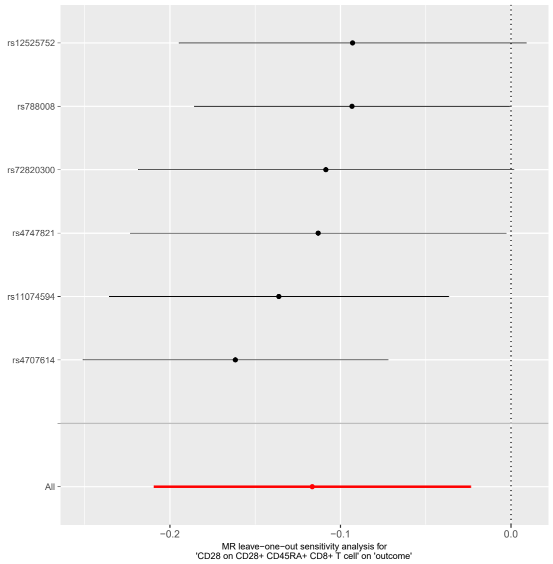
rs12525752
rs788008
rs72820300
rs4747821
rs11074594
rs4707614
All
−0.2
−0.1
0.0
MR leave−one−out sensitivity analysis for
'CD28 on CD28+ CD45RA+ CD8+ T cell' on 'outcome'

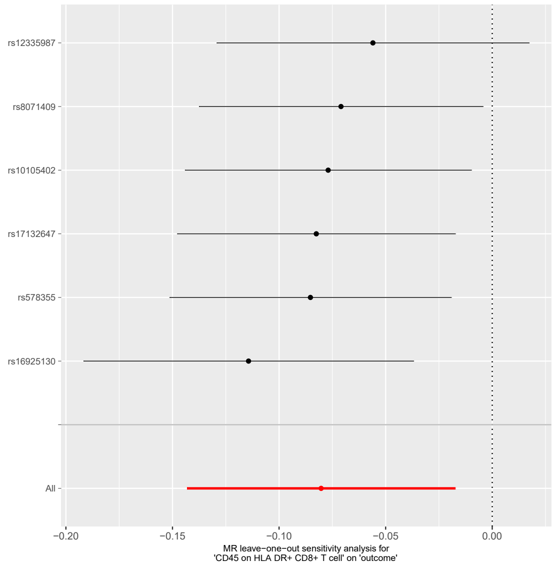
rs12335987
rs8071409
rs10105402
rs17132647
rs578355
rs16925130
All
−0.20
−0.15
−0.10
−0.05
0.00
MR leave−one−out sensitivity analysis for
'CD45 on HLA DR+ CD8+ T cell' on 'outcome'

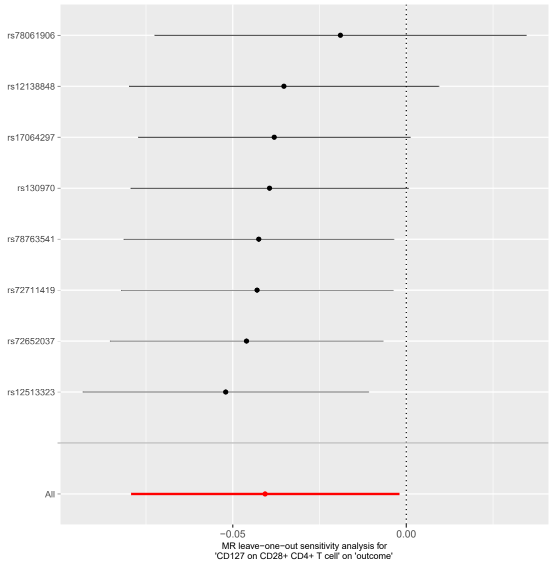
rs78061906
rs12138848
rs17064297
rs130970
rs78763541
rs72711419
rs72652037
rs12513323
All
−0.05
0.00
MR leave−one−out sensitivity analysis for
'CD127 on CD28+ CD4+ T cell' on 'outcome'

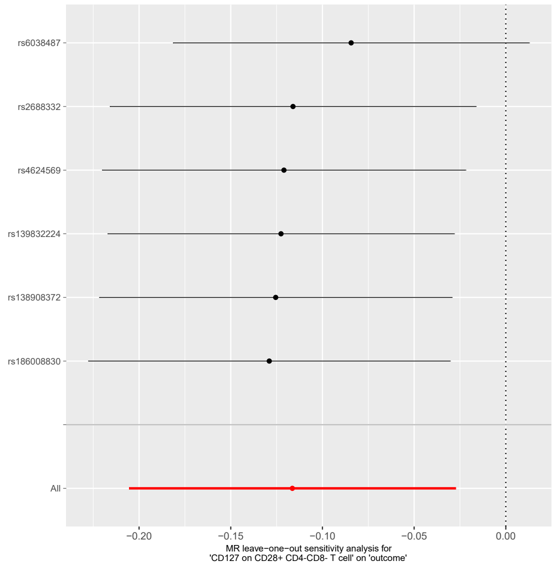
rs6038487
rs2688332
rs4624569
rs139832224
rs138908372
rs186008830
All
−0.20
−0.15
−0.10
−0.05
0.00
MR leave−one−out sensitivity analysis for
'CD127 on CD28+ CD4-CD8- T cell' on 'outcome'

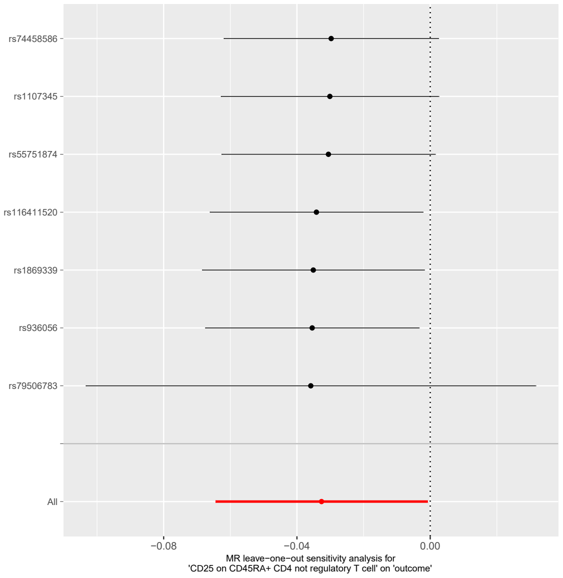
rs74458586
rs1107345
rs55751874
rs116411520
rs1869339
rs936056
rs79506783
All
−0.08
−0.04
0.00
MR leave−one−out sensitivity analysis for
'CD25 on CD45RA+ CD4 not regulatory T cell' on 'outcome'

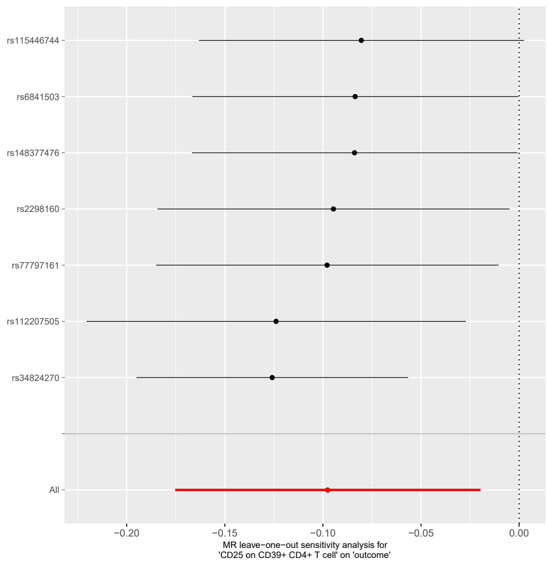
rs115446744
rs6841503
rs148377476
rs2298160
rs77797161
rs112207505
rs34824270
All
−0.20
−0.15
−0.10
−0.05
0.00
MR leave−one−out sensitivity analysis for
'CD25 on CD39+ CD4+ T cell' on 'outcome'

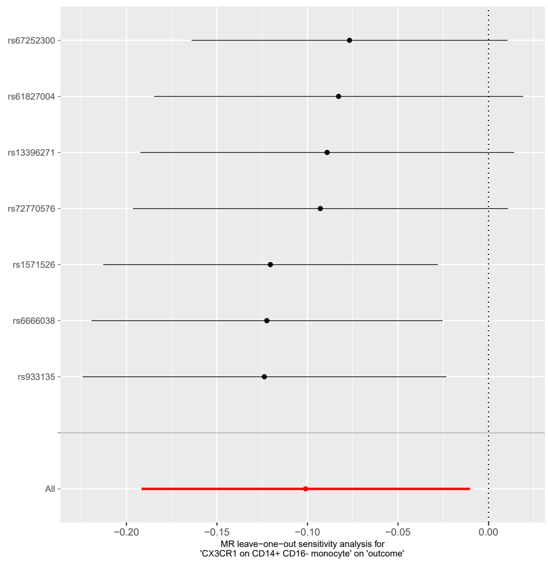
rs67252300
rs61827004
rs13396271
rs72770576
rs1571526
rs6666038
rs933135
All
−0.20
−0.15
−0.10
−0.05
0.00
MR leave−one−out sensitivity analysis for
'CX3CR1 on CD14+ CD16- monocyte' on 'outcome'

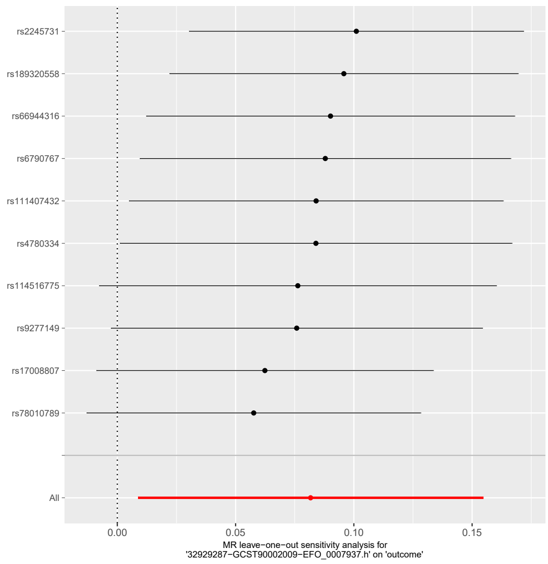
rs2245731
rs189320558
rs66944316
rs6790767
rs111407432
rs4780334
rs114516775
rs9277149
rs17008807
rs78010789
All
0.00
0.05
0.10
0.15
MR leave−one−out sensitivity analysis for
'32929287−GCST90002009−EFO_0007937.h' on 'outcome'

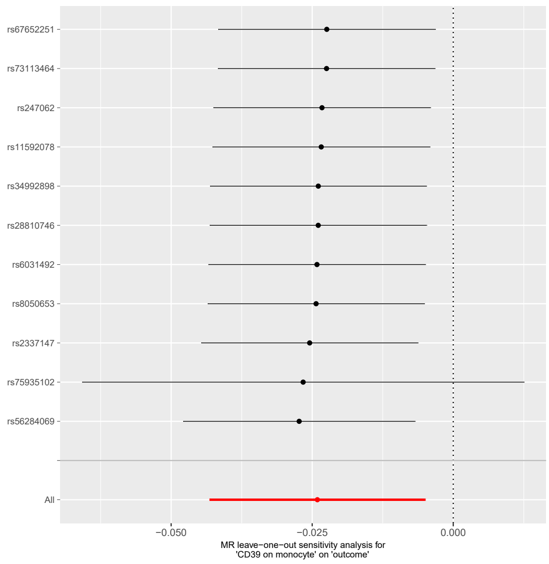
rs67652251
rs73113464
rs247062
rs11592078
rs34992898
rs28810746
rs6031492
rs8050653
rs2337147
rs75935102
rs56284069
All
−0.050
−0.025
0.000
MR leave−one−out sensitivity analysis for
'CD39 on monocyte' on 'outcome'

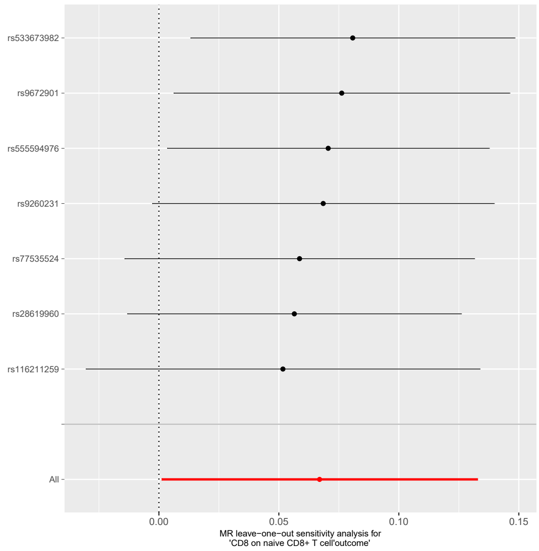
rs533673982
rs9672901
rs555594976
rs9260231
rs77535524
rs28619960
rs116211259
All
0.00
0.05
0.10
0.15
MR leave−one−out sensitivity analysis for
'CD8 on naive CD8+ T cell'outcome'

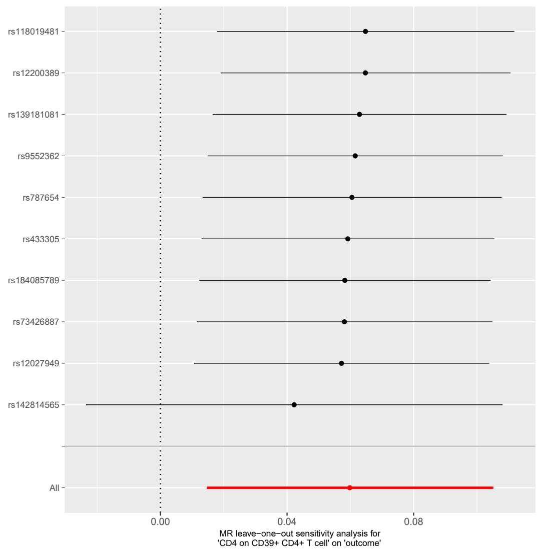
rs118019481
rs12200389
rs139181081
rs9552362
rs787654
rs433305
rs184085789
rs73426887
rs12027949
rs142814565
All
0.00
0.04
0.08
MR leave−one−out sensitivity analysis for
'CD4 on CD39+ CD4+ T cell' on 'outcome'

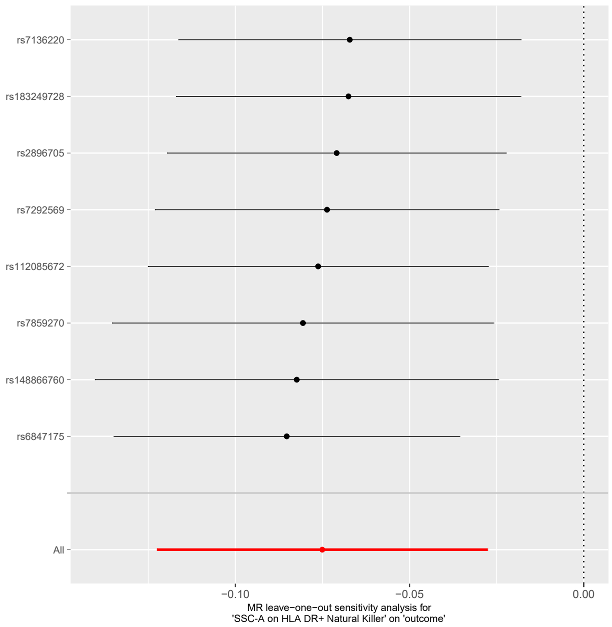
rs7136220
rs183249728
rs2896705
rs7292569
rs112085672
rs7859270
rs148866760
rs6847175
All
−0.10
−0.05
0.00
MR leave−one−out sensitivity analysis for
'SSC-A on HLA DR+ Natural Killer' on 'outcome'

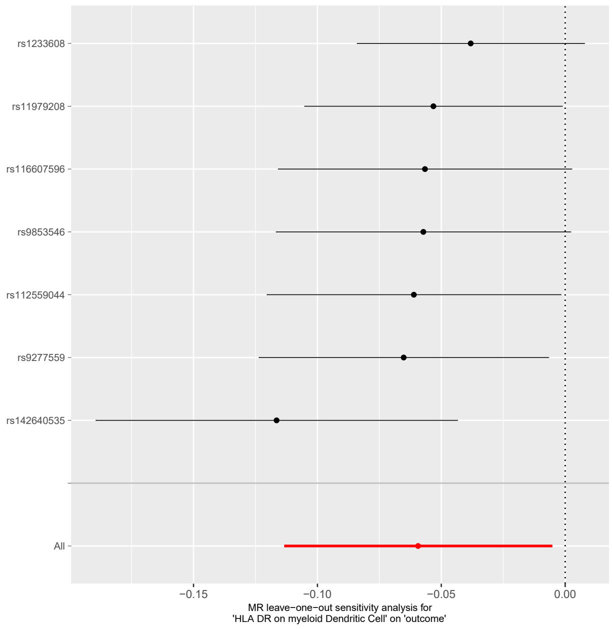
rs1233608
rs11979208
rs116607596
rs9853546
rs112559044
rs9277559
rs142640535
All
−0.15
−0.10
−0.05
0.00
MR leave−one−out sensitivity analysis for
'HLA DR on myeloid Dendritic Cell' on 'outcome'
